# Analysis of chromatin accessibility in *p53* deficient spermatogonial stem cells for high frequency transformation into pluripotent state

**DOI:** 10.1111/cpr.13195

**Published:** 2022-02-04

**Authors:** Sitong Liu, Rui Wei, Hongyang Liu, Ruiqi Liu, Pengxiao Li, Xiaoyu Zhang, Wei Wei, Xiaodong Zhao, Xiaomeng Li, Yang Yang, Xueqi Fu, Kang Zou

**Affiliations:** ^1^ 12510 College of Life Sciences Jilin University Changchun China; ^2^ 70578 Germline Stem Cells and Microenvironment Lab College of Animal Science and Technology Nanjing Agricultural University Nanjing China; ^3^ 12474 Key Laboratory of Systems Biomedicine (Ministry of Education) Shanghai Center for Systems Bio‐medicine Shanghai Jiao Tong University Shanghai China; ^4^ 47821 The Key Laboratory of Molecular Epigenetics of MOE Institute of Genetics and Cytology Northeast Normal University Changchun China; ^5^ 12461 State Key Laboratory of Reproductive Medicine Nanjing Medical University Nanjing China

**Keywords:** ATAC‐seq, chromatin accessibility, embryonic stem‐like, germline, transcription factor

## Abstract

**Objectives:**

Spermatogonial stem cells (SSCs), the germline stem cells (GSCs) committed to spermatogenesis in niche, can transform into pluripotent state in long‐term culture without introduction of exogenous factors, typically in *p53* deficiency condition. As the guardian for genomic stability, p53 is associated with epigenetic alterations during SSCs transformation. However, the mechanism is still unknown, since complicated roles of p53 baffle our understanding of the regulating process.

**Materials and Methods:**

The chromatin accessibility and differentially expressed genes (DEGs) were analysed in *p53* ^+/+^ and *p53* ^−/−^ SSCs using the Assay for Transposase‐Accessible Chromatin with high‐throughput Sequencing (ATAC‐seq) and RNA‐sequencing (RNA‐seq), to explore the connection of p53 and cell fate at chromosomal level.

**Results:**

Several transcription factors (TFs), such as CTCF, SMAD3 and SOX2, were predicted as important factors mediating the transformation. Molecular evidence suggested that SMAD3 efficiently promoted pluripotency‐associated gene expression both in fresh and long‐term cultured SSCs. However, *p53* knockout (KO) is insufficient to induce SMAD3 expression in SSCs.

**Conclusions:**

These observations indicate that SMAD3 is a key factor for SSCs transformation, and an unknown event is required to activate SMAD3 as the prerequisite for SSCs reprogramming, which may occur in the long‐term culture of SSCs. This study demonstrates the connection of *p53* and pluripotency‐associated factors, providing new insight for understanding the mechanisms of SSCs reprogramming and germline tumorigenesis.

## Graphical abstract

This study unveils that *p53* deficiency is insufficient for spermatogonial stem cells (SSCs) to obtain pluripotency during long‐term culture, and activation of SMAD3 is a key event required for SSCs transformation, which combined with *p53* deficiency has a synergic effect in mediating transformation. These observations answered why *p53* deficiency accelerated SSCs transformation and facilitated us for further understanding of the mechanism of reprogramming and tumorigenesis in germline cells.

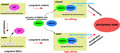

## 1 INTRODUCTION

Testicular germ cell tumours (TGCTs) are rare among pediatric ages, making up 0.5% of pediatric malignancies, but rise to 14% in adolescent malignancies, and become the most common solid tumour in young adults, representing 0.4% of new cases from all sites.^1^ The incidence rate of testicular germ cell tumours starts to increase in the late teens (10 years old) and reaches its peak in the young adult age group.^1^ The underlying mechanism of germ cell transformation into tumour cell is not clear, yet. Notably, *p53* deficient mice have a high frequency of testicular teratoma,^2^ and clinical observations showed that p53 dysfunction is usually concomitant with enhanced expression of NANOG in TGCTs.^3^, ^4^ Therefore, TGCTs are possibly associated with p53 dysfunction and the dedifferentiation of SSCs from male puberty to adult.

SSCs are germline stem cells with capacities of self‐renewal and production of functional sperm through multi‐steps of differentiation. SSCs are believed to be unipotent when they reside in their microenvironment (also called niche), since their fates are under the control of signals from the niche.^5^ However, transformation of SSCs into embryonic stem cells‐like (ES‐like) state is occasionally observed during long‐term culture *in vitro*.^6^ The morphology of the transformed cells is distinct from a typical SSCs cluster, but very similar to embryonic stem cells (ESCs) colony. Moreover, the expression of germline markers is hardly detected. Instead, the pluripotent markers, such as *Nanog*, *Sox2*, are highly expressed in the transformed ES‐like cells. Subcutaneous injection of ES‐like cells into nude mice could form teratoma with a comparable efficiency with ESCs, which confirms their pluripotency identity.^6^

This special phenomenon is interesting and important, since the dedifferentiation process does not rely on any transgenic operation or stimulation by chemicals. In contrast to reprogramming using Yamanaka factors, the underlying mechanism of SSCs transformation is still ambiguous. *Oct4*, a Yamanaka factor essential for pluripotency, is ubiquitously expressed in germline, including primordial germ cells (PGCs), SSCs, female germline stem cells (FGSCs) and oocytes.^7^, ^8^ However, the endogenous expression level of *Oct4* is relatively low in wild type of SSCs compared to ESCs or transformed SSCs, according to published studies^9^ and observation in our laboratory. Enhanced *Oct4* expression is essential for the transformation of primed ESCs to higher hierarchy, naïve state, which indicates that the alteration of *Oct4* expression level may play a key role in SSCs transformation.^10^ Moreover, Shinohara and his colleagues noticed that the loss of *p53* improved the transformation efficiency of SSCs.^6^ They further revealed that epigenetic modification played an important role in SSCs transformation, and explained that *p53* deficiency rescued SSCs from extensive cell apoptosis during transformation induced by the rewriting of DNA methylation profiles in SSCs.^11^ However, they also commented that the underlying mechanism was more complicated than that, since knockdown of *Bax* failed to promote SSCs transformation into pluripotent state.^11^ Notably, the activity of *p53* has been identified as an effective factor for cell reprogramming,^12^, ^13^ since activated p53 could suppress the expression of *Nanog*, a key pluripotent gene,^14^ and p53 is pivotal in maintenance of the genomic stability.^15^ Therefore, p53 is believed as a key bottleneck for reprogramming,^12^, ^16^, ^17^ since overexpression of the reprogramming factor (OCT4, SOX2, KLF4 and c‐MYC, which are oncoproteins) always activates *p53* to cause cell cycle arrest, apoptosis and senescence, and simultaneously suppresses the expression of *Nanog* in somatic cells.^18^

Based on these observations, we proposed that the impact of *p53* deficiency on chromatin accessibility is pivotal to elucidate the mechanism of p53 in the suppression of pluripotency transformation. However, it is complicated to reveal the exact roles of p53 in reprogramming, since p53 targets on multiple regions of chromosomes. In recent years, Assay for Transposase‐Accessible Chromatin with high‐throughput Sequencing (ATAC‐seq) has been developed to explore the link between chromatin accessibility and biological phenomenon.^19^ By analysing the open regions of chromatin, the genomic regions with altered chromatin accessibility could be profiled and allow the identification of potential transcriptional regulators involved in cellular reprogramming.^20^

Here, we employed ATAC‐seq to compare the difference of transcription active regions in the chromatin of *p53* ^+/+^ and *p53* ^−/−^ SSCs, to explore the underlying connection between the *p53* deficiency and transformation into pluripotent state at chromosomal level. RNA‐seq and molecular assays were subsequently exerted to verify the predicted genes and related pathways associated with SSCs transformation. This result enhances our further understanding of the connection of chromatin accessibility mediated by *p53* and SSCs fates, which provides a new insight into the prevention and curing of testicular tumours.

## 2 MATERIALS AND METHODS

### 2.1 Mice

The *p53* ^−/−^ transgenic allele‐carrying mice were purchased from the Shanghai Model Organisms Center, and C57BL/6 mice were supplied by Yangzhou University. For the genotyping of *p53* ^−/−^ mice, genomic DNA samples extracted from mouse tail tips were used for polymerase chain reaction (PCR) as follows: The touchdown‐PCR was carried out according to the following cycling programme: 94℃ for 2 min, followed by 10 cycles at decreasing annealing temperatures in decrements of 0.5℃ per cycle, 94℃ for 20 s, 65℃ for 15 s and 68℃ for 10 s, then 25 cycles of 15 s at 94℃, 15 s at 60℃, 10 s at 72℃, and final extension at 72℃ for 2 min (primer 1: TGGATGGTGGTATACTCAGAGC, primer 2: CAGCCTCTGTTCCACATACACT, primer 3: AGGCTTAGAGGTGCAAGCTG).

All animal experiments were performed according to the Animal Protection Guidelines of Nanjing Agricultural University and Jilin University.

### 2.2 SSCs purification using Fluorescence‐Activated Cell Sorting (FACS)

Testes from 5‐day‐old *p53* ^+/+^ or *p53* ^−/−^ mice were harvested for SSCs sorting using the protocol of the previous study.^21^ Briefly, tunica albuginea removed testes were sliced into small pieces and digested with collagenase IV at 37℃ in a water incubator for 20 min. After washing with D‐Hanks, the seminiferous tubule fragments were incubated with 0.05% trypsin at 37℃ for 5 min in a water incubator. After removal of the enzyme solution via centrifugation, the cell pellet was resuspended and filtered with a 70‐μm filter. The cell sample was washed with phosphate‐buffered saline (PBS) and was resuspended with FACS buffer at a concentration of 1 × 10^7^ cells/ml, followed by incubation with anti‐THY1 and anti‐c‐kit antibodies at 4°C for 0.5–1 h. After centrifugation and removal of the antibody‐containing supernatant, the cells were resuspended in FACS buffer for FACS sorting. The THY1^+^c‐kit^−^ fraction was collected for centrifugation and resuspended to a desired concentration before plated on mouse embryonic fibroblast (MEF) feeder layers. The protocol for preparing MEF was as previously described.^21^

### 2.3 Cell culture and transformation

Both *p53* ^+/+^ and *p53* ^−/−^ SSCs were able to be maintained in Shinohara's Iscove's Modified Dulbecco's Medium (IMDM)/foetal bovine serum (FBS) culture medium^22^ for 30 passages with a minor modification. Briefly, insulin, putrescine and transferrin were replaced with N_2_. Isolated SSCs were placed on MEF feeder layers and were subcultured every 5–6 days for 6–8 passages, and every 3 days later.

A few ES‐like colonies formed around 25 passages, and these colonies were picked under microscope and transferred to ESC culture medium. It took around 5–7 days for the subculture of ES‐like cells in the first several passages, and the average subculture time reduced to 3 days after 10 passages. The components of modified Shinohara's GSC medium and ESC medium are summarized in Table S1.

### 2.4 SSCs labelling and transplantation

SSCs cultured on MEF for more than 12 passages were infected with green fluorescent protein (GFP) expressing lentivirus. The lentivirus package was identical to that used in a previous study.^23^ The uninfected SSCs were eliminated with puromycin, and the injection procedure followed the reported protocol^24^ with minor modification: the GFP‐labelled SSCs were digested into single‐cell suspension and filtered with a 70‐μm filter, and trypan blue was added to monitor the cell injection efficiency.

Immunofluorescence (IF) staining, alkaline phosphatase (AP) staining and Western blotting.

The protocol for IF assay was identical to that given in a previous study.^25^ Briefly, cells were fixed with Carnoy for 20 min at −20℃ and were rinsed with neutral PBS for three times before blocking with 10% goat serum for 30 min at room temperature. Cells were incubated with primary antibodies at 4℃ overnight and were incubated with appropriate secondary antibodies for 1 h after rinse. Finally, DAPI (4′,6‐diamidino‐2‐phenylindole) was used for counterstaining.

The BCIP/NBT (5‐bromo‐4‐chloro‐3‐indolyl‐phosphate/nitro blue tetrazolium)/alkaline phosphatase staining kit (Beyotime, C3206) was used to detect alkaline phosphatase activity. Briefly, ESCs, ES‐like cells from *p53* **^+/+^** or *p53* **^−/−^** SSCs and primary SSCs were rinsed with PBS and incubated with BCIP/NBT solution for 30 min in dark. After the removal of BCIP/NBT solution, the cell samples were rinsed with Millipore H_2_O to terminate staining, and finally were analysed under the microscope.

The protocol for Western blotting identical to previously described^21^ was briefly listed: protein lysates were separated with sodium dodecyl sulphate‐polyacrylamide (SDS‐PAGE) gels, and the gels were transferred to nitrocellulose membranes for blotting. Nitrocellulose membranes were blocked in 5% milk for 1 h prior to the addition of primary antibody at 4℃ overnight and then were rinsed twice with TBST (Tris‐buffered saline with Tween 20). Peroxidase‐conjugated goat anti‐rabbit immunoglobulin G (IgG) or goat anti‐mouse IgG was used to detect the primary antibodies. Immunoreactive bands were visualized using the enhanced chemiluminescence (ECL) and exposed to the film. The intensity of the bands was quantified using the ImageJ software.

The information of antibodies used for IF and Western blot is listed in Table S2.

### 2.5 Reverse transcription‐polymerase chain reaction (RT‐PCR)

For reverse transcription, total RNA extracted from SSCs with TRNzol (Tiangen, DP424) was converted into complementary DNA (cDNA) using GoScript™ Reverse Transcription System (Promega, A5001). Subsequently, PCR was performed using Premix Ex Taq (Takara). The information of primers is listed in Table S3.

### 2.6 Transfection

The pcDNA3‐FLAG‐*Smad3* expression vector was constructed, as described previously.^26^ The protocols for transfection assays were identical to those used in a previous study.^25^ Briefly, for the overexpression of SMAD3, SSCs after 20 passages were transfected with pcDNA3‐FLAG‐*Smad3* or empty vector using Lipofectamine 3000 (Thermo Fisher, L3000015), and the samples were harvested 48 h posttransfection for Western blot analysis. Similarly, the scrambled or *Smad3* siRNA (5'‐GAGAUUCGAAUGACGGUAATT‐3′ ^27^) was transfected into newly isolated or long‐term cultured SSCs using Lipofectamine 3000, and the cells were harvested 48 h posttransfection for gene expression analysis.

### 2.7 ATAC‐seq and data analysis

THY1^+^ and c‐Kit^−^ cells were collected from *p53* ^+/+^ and *p53* ^−/−^ mice using flow cytometry and amplified on MEF for 5–6 passages. Around 1x10^5^ cells were collected for the ATAC‐seq assay. ATAC‐seq library preparation and sequencing were performed according to previously described.^28^ All paired‐end reads were first subjected to adaptor trimming using cutadapt (v2.10). Then, the clipped reads were aligned to the mouse genome (10 mm) using bowtie2 (v2.3.3.1) with the parameters: ‐t ‐q ‐N 1 ‐L 25 ‐X 2000 no‐mixed nodiscordant.^29^ PCR amplicon duplicates were then removed using PicardTools (v2.2.4). For downstream analysis, nonuniquely mapped reads or reads mapped to the mitochondrial genome, Y chromosome and unmapped contigs were removed.^30^ To visualize the ATAC‐seq signal in Integrative Genomics Viewer (IGV, v2.5.3), bam files were converted to bigwig (BW) files using deeptools (v3.3.0). Peaks were called for each sample using MACS2 (v2.2.6) in narrowPeak mode. Peaks overlapping with encoded backlist regions (http://hgdownload.cse.ucsc.edu/goldenPath/hg19/encodeDCC/wg 5EncodeMapability/wgEncodeDacMapabilityConsensusExcludable. bed.gz) were removed using bedtools (v2.26.0–148‐gd1953b6). We performed differential chromatin accessibility (DA) analysis using the DiffBind package with the DESeq2 algorithm. DA was defined with the criteria of a false discovery rate (FDR) <0.05 and absolute log2 (fold change) ≥1. To identify TF motifs in *p53* KO‐enriched peaks enriched in *p53* KO SPCs, a homer^31^ (v4.10.4) *de novo* motif analyser was used by setting parental enriched peaks as the background.^28^, ^32^ Gene ontology (GO) and Kyoto Encyclopedia of Genes and Genomes (KEGG) analysis were performed with the Database for Annotation, Visualization and Integrated Discovery (DAVID).

The original data of ATAC‐seq assay have been uploaded to https://www.ebi.ac.uk/fg/annotare/, and E‐MTAB‐10012 is the code to review the original data.

### 2.8 RNA‐seq and data analysis

Around 10^5^ *p53* ^+/+^ and *p53* ^−/−^ SSCs were collected using the identical protocols for ATAC‐seq assay. RNA‐seq library preparation and sequencing were performed according to previously described.^28^ Total RNA was extracted using Trizol (Ambion Life Technologies) according to the Ambion standard RNA isolation procedure, and messenger RNA (mRNA) was purified using the NEBNext Poly (A) mRNA Magnetic Isolation Beads (NEB, USA). Then, the mRNA library was constructed with a NEBNext Ultra Directional RNA Library Prep Kit for Illumina (E7420S/L, NEB) and sequenced with Illumina HiSeq 2000. DEGs analysis was performed to compare *p53* ^+/+^ and *p53* ^−/−^ SSCs using the DESeq2 R package. DEGs were defined with the criteria of q‐value <0.05 and absolute log (fold change) ≥1.5 (*p *< 0.03). Gene ontology (GO) and KEGG analysis were performed with DAVID.

For analysis, the connection of chromatin change and DEGs in Venn diagram, individual peaks separated by <100 bp were joined together using bedtools. Peak annotation was performed using HOMER. The duplicate genes in the RNA‐seq results have been removed for Venn analysis.

The original data of RNA‐seq assay have been uploaded to https://www.ebi.ac.uk/fg/annotare/, and E‐MTAB‐10608 is the code to review the original data.

### 2.9 Quantification and statistical analysis

Data were analysed by Excel and were presented as mean ±SD (standard deviation), and statistical significance was determined by the *t*‐*test*.

## 3 RESULTS

### 3.1 Collection and verification of SSCs from *p53* deficient mouse

**FIGURE 1:**
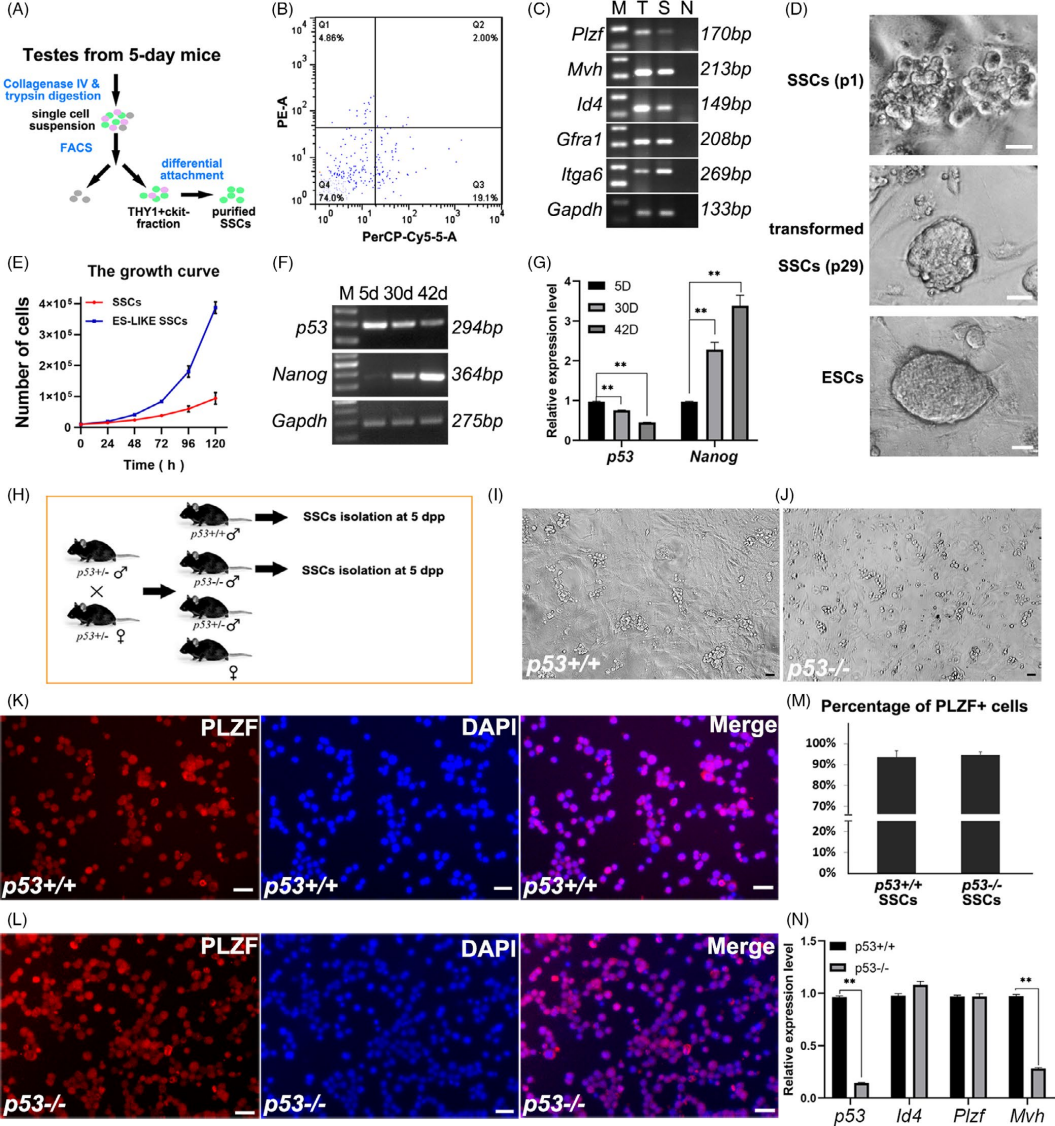
Identification of spermatogonial stem cells (SSCs) from *p53* ^+/+^ or *p53* ^−/−^ mice. The schematic illustration of SSCs sorting is exhibited (A), and a representative of SSCs sorting using fluorescence‐activated cell sorting (FACS) (PE: c‐kit, PerCP: THY1) (B). Sorted cells were identified for SSCs markers using reverse transcription‐polymerase chain reaction (RT‐PCR), M. marker, T. testis, S. SSCs, N. negative control (C). The morphologies of SSCs of the first passage, transformed embryonic stem cells‐like (ES‐like) cells (29 passages) and embryonic stem cells (ESCs) colonies is exhibited (D). The growth curves of SSCs and ES‐like are exhibited (E). SSCs sorted from 5‐day‐, 30‐day‐ and 42‐day‐old mice were determined for *p53* and *Nanog* expression using RT‐PCR (F), and the results were statistically exhibited (*n* = 3, *p < 0.05, **p < 0.01) (G). A representative result of genotyping for the litters at 3 days postpartum using PCR (H). The SSCs sorted from 5‐day‐old *p53* ^+/+^ (I) or *p53* ^−/−^ mice (J) were cultured on mouse embryonic fibroblast (MEF), and the morphology was exhibited. Immunostaining of promyelocytic leukaemia zinc finger (PLZF) was used to verify SSCs from *p53* ^+/+^ (K) or *p53* ^−/−^ mice (L), and the percentage of PLZF^+^ cells was statistically analysed (M). RT‐PCR was used to detect the expression of *p53*, *Id4*, *Plzf*, *Mvh* and *Gapdh* in SSCs from *p53* ^+/+^ and *p53* ^−/−^ mice (n=3) (N). The data represent the means ± SD (*p < 0.05; **, *p* < 0.01). Scale bar = 20 µm

Based on several protocols that can efficiently enrich SSCs and achieve SSCs long‐term culture,^22^, ^33^, ^34^ we sorted SSCs (Figure 1 A‐B). They express undifferentiated spermatogonia markers (Figure 1C) and SSCs marker ID4 (Figure S1A–C), could be maintained on MEF feeder layer for more than 30 passages in IMDM/FBS condition^22^ and restore the capacity to reconstitute the fertility of busulfan‐treated mice (Figure S1D–F). Interestingly, we frequently observed that a few SSCs always transformed into ES‐like state around 25 passages under this culture condition, and these colonies could be stably maintained on MEF feeder layers (Figure 1D) with a higher proliferation ratio than untransformed SSCs (Figure 1E). SSCs spontaneous reprogramming was reported in 2004,^6^ despite the fact that their culture medium was slightly different. Shinohara and his colleagues also reported that the *p53* deficiency remarkably increased the transformation efficiency,^6^ and indicated that this event was associated with epigenetic change caused by *p53* loss.^11^ Therefore, we focused on the role of *p53* in regulating SSCs fate, to further demonstrate the molecular mechanism of SSCs reprogramming, and to reveal the connection of *p53* expression with age and SSCs fate. First, SSCs isolated from 5‐day‐, 30‐day‐ and 42‐day‐old mice were examined using RT‐PCR, to track the expression change of *p53* in SSCs of neonatal, juvenile and young adult testes. The expression level of *p53* in SSCs of 30‐day testes decreased by 35% compared to that in SSCs of 5‐day testes, and decreased by about 60% in SSCs harvested from 42‐day testes (Figure 1F,G). On the contrary, expression of *Nanog* remarkably increased with age at mRNA level (Figure 1F,G). Expression of NANOG was not detected in SSCs at protein level using Western blot (data not shown). These observations suggested that the expression of *p53* and the potential of pluripotency transformation may increase in SSCs during ageing. Considering that *p53* loss promotes the spontaneous reprogramming of SSCs into pluripotent state, we hypothesize that the increased rate of tumorigenesis in adolescent and adult testes is possibly associated with *p53* loss or dysfunction in SSCs.

To understand the potential mechanism of increased transformation efficiency caused by *p53* deficiency, we harvested SSCs from testes of 5‐day‐old *p53* ^+/+^ and *p53* ^−/−^ mice for investigation (Figure 1H). Testes were digested to single‐cell suspension for SSCs sorting using FACS, and THY1^+^c‐kit^−^ populations were collected for identification, and both *p53* ^+/+^ and *p53* ^−/−^ SSCs formed typical SSCs clusters (Figure 1I,J). The purity of sorted cells was determined using promyelocytic leukaemia zinc finger (PLZF) (the marker of undifferentiated spermatogonia) IF staining (Figure 1K,L), and statistical analysis revealed that the PLZF^+^ ratio is approximate to 93% (93.6 ± 2.9% in *p53*+/+ SSCs and 94.6 ± 1.4% in *p53* ^−/−^ SSCs) (Figure 1M). The expression levels of *p53*, SSCs markers *Id4* and *Plzf*, germline marker *Mvh* were determined using RT‐PCR, which revealed identical expression levels of SSCs markers *Id4* and *Plzf* in *p53* ^+/+^ and *p53* ^−/−^ SSCs, but *Mvh* expression was remarkably decreased (Figure 1N). Therefore, we confirmed that SSCs‐enriched populations were collected, and *p53* ^+/+^ and *p53* ^−/−^ SSCs were indistinguishable for their morphological characteristics, but the expression of molecular markers was probably not identical.

### 3.2 Verification of the transformation capacity of *p53* deficient SSCs

**FIGURE 2:**
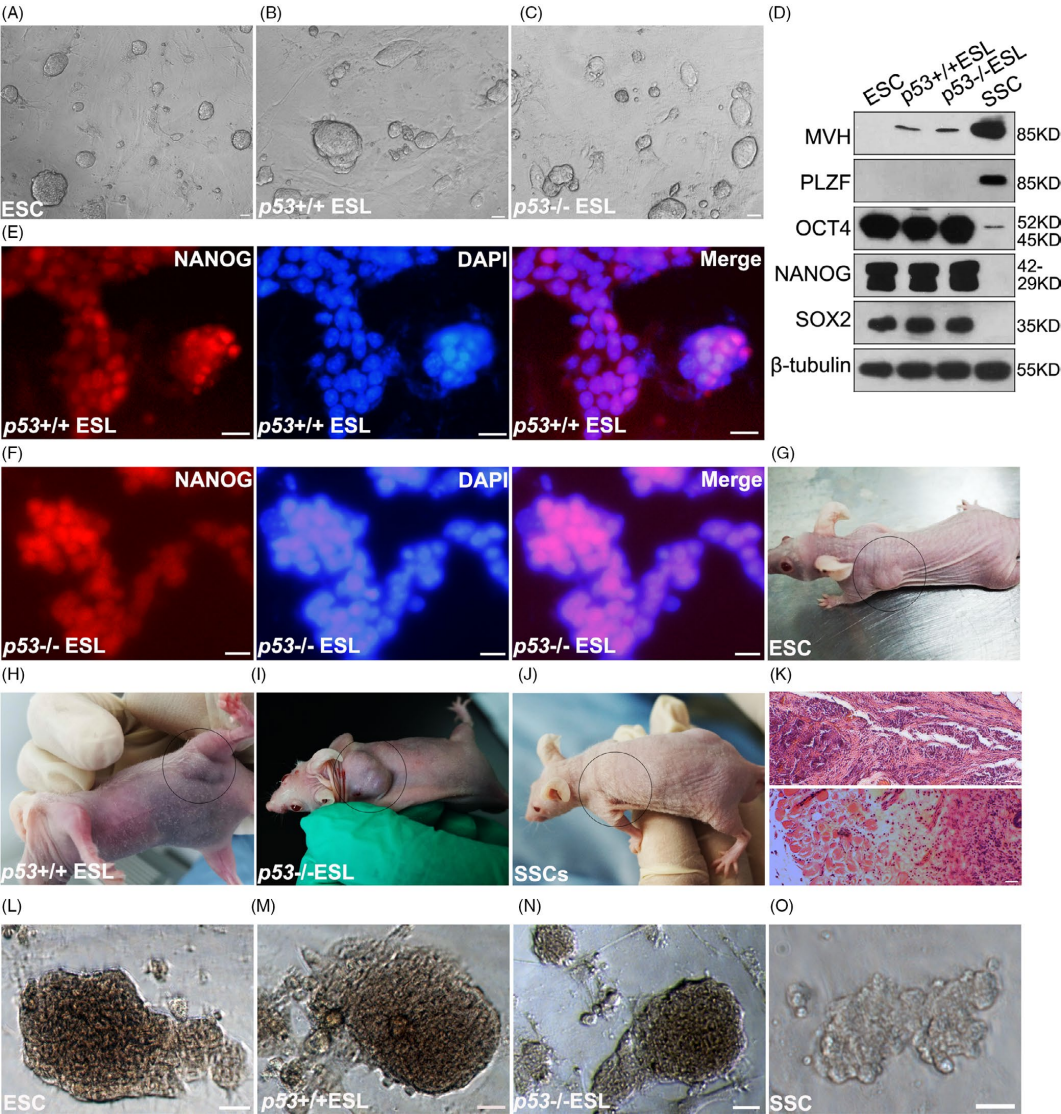
Verification of the transformation of *p53* ^+/+^ and *p53* ^−/−^ SSCs (spermatogonial stem cells) into pluripotent state. The morphologies of embryonic stem cells (ESCs) (A), embryonic stem cells (ES)‐like cells derived from *p53* ^+/+^ (B) or *p53* ^−/−^ SSCs (C) maintained on mouse embryonic fibroblast (MEF) feeder layers were exhibited. The expression levels of germline markers (mouse vasa homologue [MVH], promyelocytic leukaemia zinc finger [PLZF]) and pluripotent markers (OCT4, NANOG, SOX2) were detected in ESCs, ES‐like cells derived from *p53* ^+/+^ SSCs, *p53* ^−/−^ SSCs, or untransformed SSCs using Western blot (*n* = 3) (D). The expression of NANOG was detected in ES‐like cells derived from *p53* ^+/+^ (E) and *p53* ^−/−^ SSCs (F) using immunofluorescence (IF) staining. Nude mice subjected to subcutaneous injection of ESCs (G), ES‐like cells derived from *p53* ^+/+^ SSCs (H), or *p53* ^−/−^ SSCs (I), and SSCs (J) subcutaneous injection were checked for teratoma (*n* = 12). A representative histology of the teratoma from *p53* ^−/−^ ES‐like cells injected mice was analysed using haematoxylin and eosin (HE) staining (K). The alkaline phosphatase (AP) staining of ESCs (L), *p53* ^+/+^ ESL (endothelial surface layer) (M), *p53* ^−/−^ ESL (N) and SSCs (O) is exhibited. Scale bar = 20 µm

Before exploring the transformation mechanism, we tested the transformation capacity of *p53* deficient SSCs under *in vitro* condition. According to the protocol (Method/Cell culture and transformation, and Table S1), both *p53* ^+/+^ and *p53* ^−/−^ SSCs were able to form ES‐like state before 30 passages, and they formed typical ES‐like colonies when transferred into ESC medium (Figure 2A–C), which was consistent with reported observations.^6^ Moreover, the expression levels of germline and pluripotent markers were also altered: high expression levels of pluripotent marker genes, including OCT4, NANOG and SOX2, were detected in both ESCs and ES‐like cells derived from *p53* ^+/+^ and *p53* ^−/−^ SSCs, confirming the transformation into pluripotent state. The undifferentiated spermatogonia marker PLZF was only detected in untransformed SSCs, and a very low level of germline marker mouse vasa homologue (MVH) was detected in ES‐like cells derived from *p53* ^+/+^ and *p53* ^−/−^ SSCs (Figure 2D), indicating that the expression profile of transformed ES‐like cells was similar to that of pluripotent cells, despite the fact that a few of the germ cells still remained in the cell mixture. Meanwhile, NANOG IF staining was employed to further test the purity of transformed cells, and the NANOG^+^ ratio was approximate to 100% in ES‐like cells derived from *p53* ^+/+^ or *p53* ^−/−^ SSCs (Figure 2E,F). Finally, ESC, ES‐like cells derived from *p53* ^+/+^ and *p53* ^−/−^ SSCs and untransformed SSCs were subcutaneously injected into nude mice for teratoma assay. Teratomas were observed in recipients of ESCs, *p53* ^+/+^ and *p53* ^−/−^ ES‐like cells, but not in SSCs recipients (Figure 2G–J), and the histology of teratoma was analysed (Figure 2K). Moreover, strong alkaline phosphatase signals were detected in ESCs, *p53* ^+/+^ and *p53* ^−/−^ ES‐like cells (Figure 2L–N), but not in SSCs after 10 passages (Figure 2O). These observations suggested that *p53* ^+/+^ and *p53* ^−/−^ SSCs have transformed into pluripotent state, which confirmed the spontaneous reprogramming capacity of SSCs.

### 3.3 The landscape of chromatin accessibility

**FIGURE 3:**
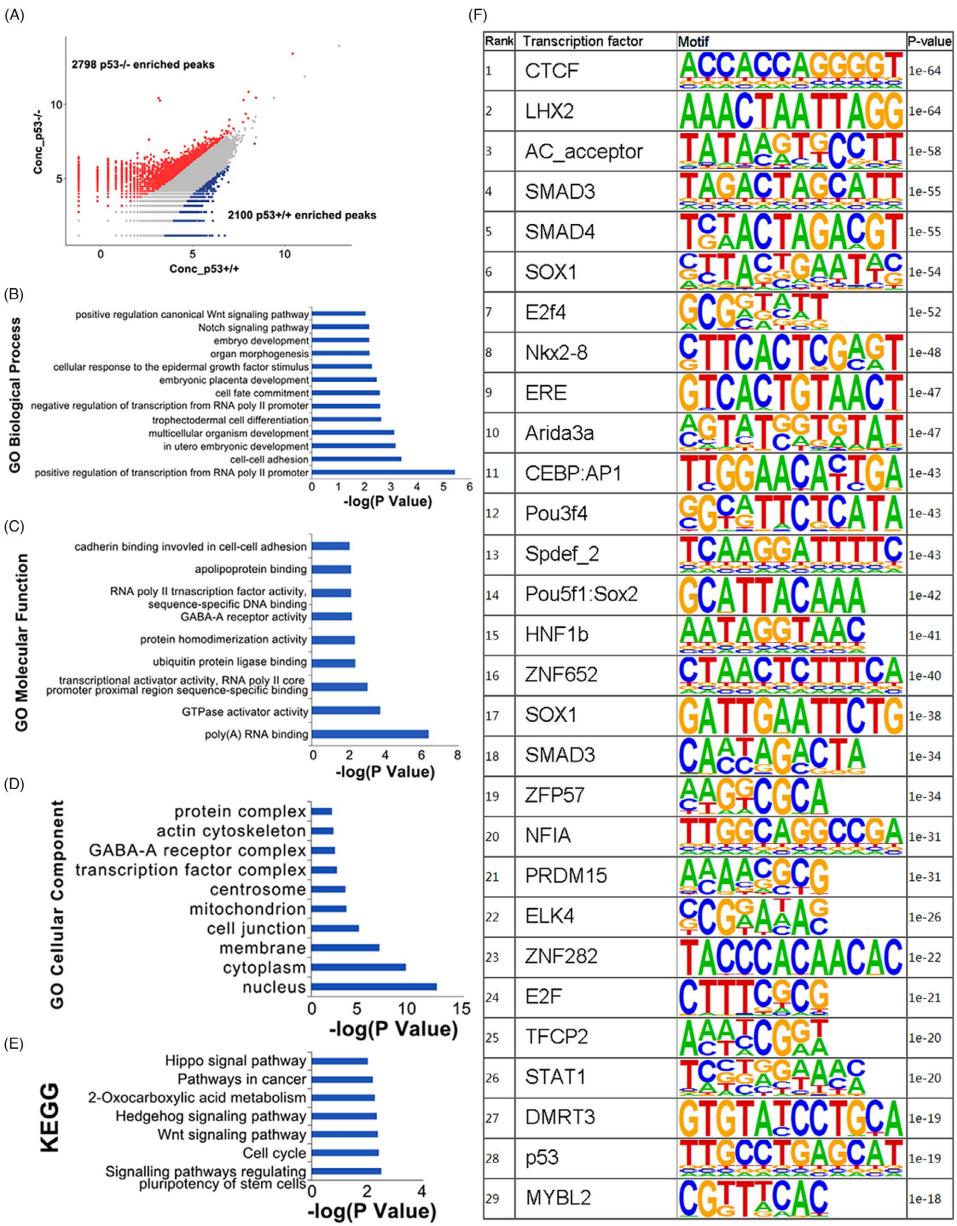
Analysis of differential chromatin accessibility (DA) in *p53* ^+/+^ and *p53* ^−/−^ SSCs (spermatogonial stem cells). Volcano plot of the differential Assay for Transposase‐Accessible Chromatin with high‐throughput Sequencing (ATAC‐seq) peak analysis between *p53* ^+/+^ and *p53* ^−/−^ SSCs (A). Gene ontology (GO) analysis exhibited the increased accessibility in genes according to the Biological Process analysis (B), Molecular Function (C) and Cellular Component (D) in *p53* ^−/−^ SSCs compared to *p53* ^+/+^ SSCs. The differential chromatin accessibility of genes was further analysed by Kyoto Encyclopedia of Genes and Genomes (KEGG) for their Biological Functions (E). The difference of binding motifs in *p53* ^+/+^ and *p53* ^−/−^ SSCs screened in the chromosomes, and the transcription factors that recognize these binding motifs, are listed (F)

Previous studies revealed the connection of epigenetic events and SSCs transformation into pluripotent state,^11^ but the exact mechanism, especially the role of p53, is still largely unknown. Here, we confirmed SSCs’ identity and their transformation potential during long‐term culture, and subsequently harvested SSCs from *p53* ^+/+^ and *p53* ^−/−^ neonatal mice for ATAC‐seq analysis, to detect the change of genomic chromatin accessibility caused by *p53* deficiency. The ATAC‐seq data from *p53* ^+/+^ and *p53* ^−/−^ SSCs were generated, and differential chromatin accessibility patterns were exhibited by the volcano map. Totally, 4,898 differentially accessible peaks were identified in *p53* ^−/−^ SSCs when comparing its wild‐type (WT) counterpart (FDR <0.05 and fold change ≥2) (Table S4, to obtain the details via this link: https://figshare.com/s/2663b87fd48c819c3ab9). Among them, 2798 regions with increased openness and 2100 areas with decreased openness were detected in *p53* **^−/−^** SSCs (Figure 3A). First, we noticed that accessibility of several pluripotency‐associated genes was increased, including *Nanog*, *Sox2*, *Mycn* and *Tgfb1* (Figure S2), confirming the link of p53 and expression of pluripotent genes. Subsequently, the genes with increased chromatin accessibility in *p53* **^−/−^** SSCs were analysed using the gene ontology (GO) analysis (*p* value<0.05). The Biological Process items of the canonical Wnt signalling pathway, Notch signalling pathway, embryo development, cellular response to the epidermal growth factor stimulus, cell fate commitment, cell‐cell adhesion, and regulating type II RNA polymerase binding, were significantly enriched (Figure 3B). According to the Molecular Function, the genes associated with poly(A)RNA binding, GTPase activator activity, transcription activator and RNA polymerase II core promoter proximal region sequencing‐specific binding were significantly enriched (Figure 3C). From the perspective of Cellular Components, genes in the nucleus, cytoplasm and cell membrane were most significantly changed (Figure 3D). In addition, KEGG results showed that the genes with increased accessibility were mainly involved in the regulation of stem cell pluripotency, cell cycle and Wnt signalling pathway (Figure 3E). These results were consistent with the phenotypes we observed: transformed cells gained pluripotency, increased in proliferation rate and colonies became more compacted (Figure 1 and Figure 2), compared to SSCs.

In addition, we made the computational prediction of TF potentially bound in the altered chromatin regions in *p53* ^−/−^ SSCs using the *de novo* TF motifs discovery software HOMER, yielding 29 transcription factors (Figure 3F). Among them, we noticed several transcription factors related to pluripotency, including CTCF, POU5F1: SOX2, SOX1, SMAD3, SMAD4, LHX2, NFATC2, E2F and E2F4, five embryonic development–related transcription factors, ARID3A, HNF1B, ZFP57, PRDM15 and DMRT3, two cell cycle–related transcription factors, MYBL and ELK4. The binding domain recognized by p53 became more open, indicating that the transcriptional activity of its potential target sites might be increased after *p53* deletion, which was in line with our expectation, and confirmed the reliability of ATAC‐seq results. We also noticed that the most significant change in openness is CTCF, a key structural protein for the high‐order chromatin folding of pluripotent stem cells.^35^ A recent study indicated that CTCF is an insulator‐binding protein, which is considered to be a key factor in the regulation of genome structure, and is closely related to cell reprogramming.^36^ The increased accessibility of CTCF‐binding regions preliminarily indicated that SSCs tended to transform into pluripotency. Increased accessibility of domains recognized by TF POU5F1: SOX2 after *p53* deficiency also implied the potential of transformation. POU5F1, also known as OCT4, is a key transcription factor for cell reprogramming. It has been reported that OCT4 was up‐regulated by SOX2 during SSCs transformation,^11^ which coincided with our experimental results. Meanwhile, we analysed the motifs with increased accessibility in ESCs or induced pluripotent stem cells (iPSCs),^37^ and noticed that domains recognized by CTCF and OCT4 had a lower nucleosome occupancy, indicating the up‐regulated transcriptional activity of these regions. This is consistent with our ATAC‐seq data. Several members of the Pit‐Oct‐Unc (POU) family, POU2F2, POU2F3, were also detected in ESCs, while the binding domain of POU3F4 was increased in *p53* deleted SSCs (Figure 3F), implying a different mechanism of reprogramming in SSCs.

Importantly, the binding domains of SMAD3 and SMAD4, two key members of the transforming growth factor‐β (TGF‐β) signalling pathway, became more accessible, indicating that the transcriptional regulation of SMAD3 and SMAD4 on their targets may altered after *p53* deletion. SMAD protein in the TGF‐β signalling pathway is associated with the regulatory network of SOX2, OCT4 and NANOG, especially SMAD2/3, which binds with SMAD4 after activation, thus activating *Nanog* expression and promoting pluripotency transformation.^38^ In addition, the binding domain of E2F4 also became open. Under the stimulation of TGF‐β, E2F4 can form a complex with SMAD3 to enter the nucleus, and combine with SMAD4 to regulate the expression of *c*‐*Myc*.^39^ These results facilitated us to preliminarily understand the characteristics of gene expression changes in SSCs caused by *p53* deletion. Therefore, we proposed that the increase of chromosomal accessibility of the regions recognized by SMAD3 and SMAD4 was an important event that led to pluripotency transformation of SSCs after *p53* deletion.

### 3.4 The differential expression profiles of *p53* ^+/+^ and *p53* ^−/−^ SSCs in transcriptome analysis

**FIGURE 4:**
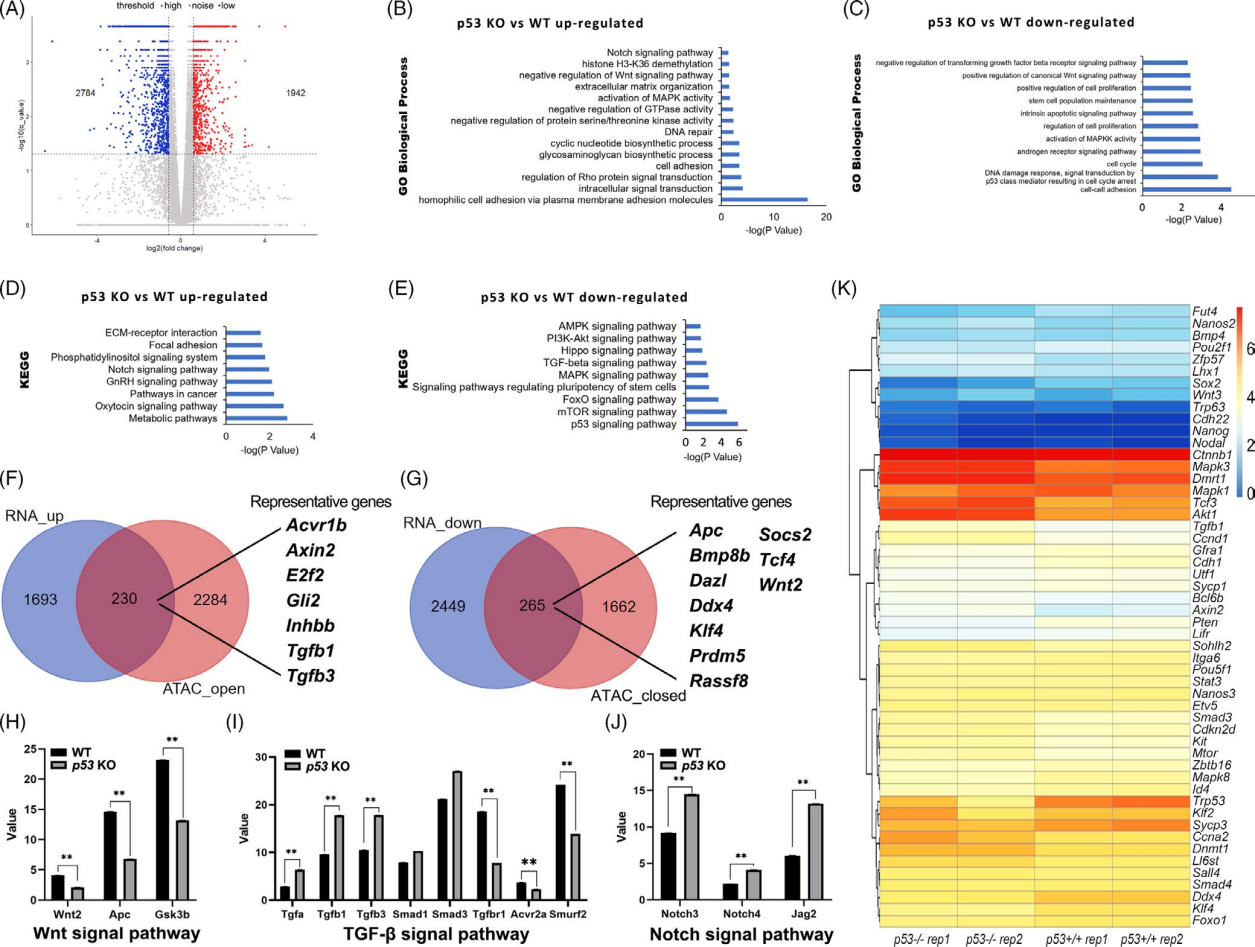
Differential pathways and genes of *p53* **^+/+^** and *p53* **^−/−^** SSCs (spermatogonial stem cells) identified by transcriptome analysis. Volcano plot of differentially expressed genes (DEGs) between *p53* ^+/+^ and *p53* ^−/−^ SSCs (A). Gene ontology (GO) analysis exhibited the up‐regulated (B) and down‐regulated (C) genes according to the Biological Process in *p53* ^−/−^ SSCs compared to *p53* ^+/+^ SSCs. The up‐regulated genes (D) and the down‐regulated genes (E) in *p53* ^−/−^ SSCs compared to *p53* ^+/+^ SSCs were analysed by Kyoto Encyclopedia of Genes and Genomes (KEGG). Venn diagram was used to exhibit the overlap of genes with up‐regulated expression levels and genes with increased chromatin accessibility (F), or genes with down‐regulated expression levels and genes with decreased chromatin accessibility (G), caused by *p53* deficiency in SSCs. The expression levels of representative genes of Wnt (H), transforming growth factor‐β (TGF‐β) (I) and Notch (J) signalling pathways in *p53* ^+/+^ and *p53* ^−/−^ SSCs are exhibited (**p‐value <0.01 ). Heatmap of genes related to pluripotency, spermatogonia, spermatocyte, cell cycle or proliferation and regulating SSCs fates in *p53* ^−/−^ SSCs and *p53* ^+/+^ SSCs (K)

To examine the differential gene expression of *p53* ^+/+^ and *p53* ^−/−^ SSCs, we performed RNA‐seq analysis. Totally, 4726 differentially expressed genes (DEGs) after *p53* deletion (q < 0.05, and absolute log (fold change) ≥1.5) were detected, including 1942 up‐regulated genes and 2784 down‐regulated genes (Table S5, to see full data, please use this link: https://figshare.com/s/a02ad4af1650888742fb). The results are displayed and analysed by the volcano map (Figure 4A).

Furthermore, the gene ontology (GO) analysis was performed with the up‐regulated or down‐regulated genes. According to the results of the Molecular Function analysis, in *p53* deleted SSCs, the expression of genes related to Notch signalling pathway, histone H3‐K36 demethylation, extracellular matrix (ECM) organization and activation of the mitogen‐activated protein kinase (MAPK) signalling pathway significantly increased, while the gene expression levels related to TGF‐β signalling pathway inhibition, canonical Wnt signal activation, cell proliferation and stem cell maintenance were significantly down‐regulated (Figure 4B,C). Subsequently, we analysed the differential expression genes in *p53* ^+/+^ and *p53* ^−/−^ SSCs using KEGG. The results showed that the up‐regulated genes in *p53* knockout SSCs included the ECM‐receptor interaction, focal adhesion, phosphatidylinositol signal system, Notch and gonadotropin‐releasing hormone (GnRH) signalling pathways, etc., while the down‐regulated genes mainly included AMP‐activated protein kinase (AMPK), PI3K‐AKT, Hippo and TGF‐β signalling pathways (Figure 4D,E). These observations were consistent with ATAC‐seq results, which revealed the increased accessibility in the genes of these signalling pathways, including Wnt, Notch, cell adhesion, etc. This indicated that *p53* affected these genes via regulating chromosomal accessibility in SSCs. Meanwhile, we focused on the pluripotent genes with increased accessibility (*Wnt10b*, *Zfhx3*, *Wnt10a*, *Wnt2b*, *Fzd2*, *Rif1*, *Dlx5*, *Esrrb*, *Pik3r1*, *Isl1*, *Bmp2*, *Kat6a*, *Akt3*, *Akt1*, *Smarcad1*, *Pcgf1*, *Nanog*, *Bmpr1b*, *Neurog1* and *Nodal*) (Figure 3E, Table S4), but most of them have not been activated, yet (Table S5). This was in accord with our expectation: *p53* deficient SSCs have not been transformed (Figure 2). Moreover, the dysregulated genes associated with change of chromatin accessibility were analysed. Deletion of *p53* led to 4898 regions with altered chromatin accessibility in SSCs, and the expression of 4726 genes was significantly affected (1.5‐fold change). By analysis of the 2798 open peaks, 2284 genes were identified, and 1693 genes were up‐regulated, after *p53* deficiency. Similarly, 1662 genes with closed chromatin state were identified from 2100 peaks, and 2449 genes were down‐regulated. About 9.76% and 11.9% altered ATAC‐seq peaks were, respectively, mapped to differentially expressed genes (DEGs) (Tables S6 and S7), matching with the range of a previous study (only 5%~12% cell type–specific ATAC‐seq peaks mapped to genes with differential expression).^40^ In the 230 up‐regulated genes whose chromosomal regions became more open (Figure 4F and Table S6), we found several genes of the TGF‐β signalling pathway (*Tgfb1*, *Tgfb3*, *Inhbbb* and *Acvr1b*) and pluripotency‐associated genes including *Axin2*, *E2f2* and *Gli2*. In the 265 down‐regulated genes with decreased accessibility after *p53* knockout (Figure 4G and Table S7), we found several germline markers, such as *Bmp8b*,^41^ *Dazl*, *Ddx4* and *Rassf8*,^42^ and genes of the Wnt signalling pathway, such as *Apc*, *Prdm5*, *Tcf4* and *Wnt2*, and pluripotency‐associated gene *Klf4*. To further dig into the molecular cues caused by *p53* loss, we specifically compared the expression changes of several pivotal genes in Wnt (Figure 4H), TGF‐β (Figure 4I) and Notch (Figure 4J) signalling pathways, since they are potentially associated with the regulation of cell fate.^43^, ^44^, ^45^ Expression levels of *Tgfa*, *Tgfb1*, *Notch3*, *Notch4* and *Jag2* were remarkably increased, and expression levels of *Apc*, *Gsk3b* and *Tgfbr1*, *Acvr2a* and *Smurf2* were remarkably decreased in *p53* deficient SSCs (q‐value <0.05 and absolute log (fold change) ≥1.5 (*p *< 0.03)) (Table S5), implying the activation of Wnt, TGF‐β and Notch signalling pathways in *p53* deficient SSCs. Based on these observations, we concluded that *p53* deletion activated Wnt, TGF‐β and Notch signalling pathways through actuating opened‐up chromatin state.

Furthermore, several genes associated with stem cell fate were chosen for analysing the expression difference in *p53* ^+/+^ and *p53* ^−/−^ SSCs, which was exhibited in the heatmap (Figure 4K). The expression levels of genes related to pluripotency, including *Bmp4*, *Oct1*, *Oct4*, *Nanog*, *Nodal*, *Sall4*, *Stat*3, *Klf2* and *Foxo1*, were not significantly changed, and the expression of *Sox2*, *Klf4* and *Utf1* was remarkably decreased, suggesting pluripotency was not obtained in *p53* deficient SSCs, yet. However, the chromosomal regions of *Nanog*, *Nodal*, *Sall4*, *Sox2* and *Foxo1* became open state (Table S4), indicating that these genes tended to be transcriptionally activated. Considering that these pluripotent‐associated genes were not activated after *p53* deletion, we speculated that these genes probably did not participate in the initiation step of transformation, or the expression was inhibited by other activated factors, for example, *Foxo1* was possibly suppressed by up‐regulated *Akt1*.^46^ Expression levels of several spermatogonia markers, including *Cdh22*, *Itga6*, *Nanos2*, *Nanos3*, *Etv5*, *Lhx1*, *Bcl6b* and *Sohlh2*, were not significantly changed, but the undifferentiated spermatogonia marker *E*‐*cadherin* (*Cdh1*), *Gfra1* and germline marker *Ddx4* were remarkably decreased. Meanwhile, the expression levels of differentiation markers including *c*‐*kit*, *Sycp1* and *Sycp2* were not remarkably changed, and *sypc3* was down‐regulated, which hinted that *p53* deficient SSCs tended to lose germline characteristics, rather than differentiate.

Notably, the binding domain of SMAD3 became open (Figure 3F), and TGF‐β signalling pathway tended to be activated (Figure 4F,I, Table S5), but neither the chromatin state nor the expression levels of *Smad3* and *Smad4* were significantly changed. On the other hand, genes of the Wnt signalling pathway, including *Wnt3*, *Axin2* and *Tcf3*, were up‐regulated, but the chromatin state and expression level of *β*‐*catenin* were not changed, either. This demonstrated that these two pathways connecting *p53* deficiency to SSCs reprogramming have not been fully activated, yet.

Tumour suppressor gene *Pten* was inhibited in *p53* deficient SSCs, and the expression levels of some cell cycle or proliferation‐associated genes *Axin2* and *Cyclin D1* were increased (Figure 4K), indicating that *p53* loss accelerates cell proliferation. Notably, the Log2(fold change) describing the expression changes of *Akt1* and *mTor* were 0.46 and 0.45 (Table S5), representing that their expression levels were about 1.37‐fold as control. They were defined as not significantly increased, since our standard was 1.5‐fold, but these differences could be observed in the heatmap (Figure 4K). Moreover, the expression of methylation‐regulated genes *Dmrt1* and *Dnmt1* was not remarkably affected (Table S5), indicating that the methylation modification mediated by these two genes^11^ has not occurred after *p53* deletion.

These results suggested that several signalling pathways associated with stem cell fate, especially TGF‐β and Wnt signalling pathways, and some pathways associated with cell cycle, epigenetic modification and DNA repair, were affected by *p53* loss. Combined with the changes of chromatin accessibility revealed by ATAC‐seq, we further speculated that *p53* deletion affected chromatin openness and activity of signalling pathways related to cell fate.

### 3.5 The relationship between chromatin accessibility and RNA‐seq measured gene expression

**FIGURE 5:**
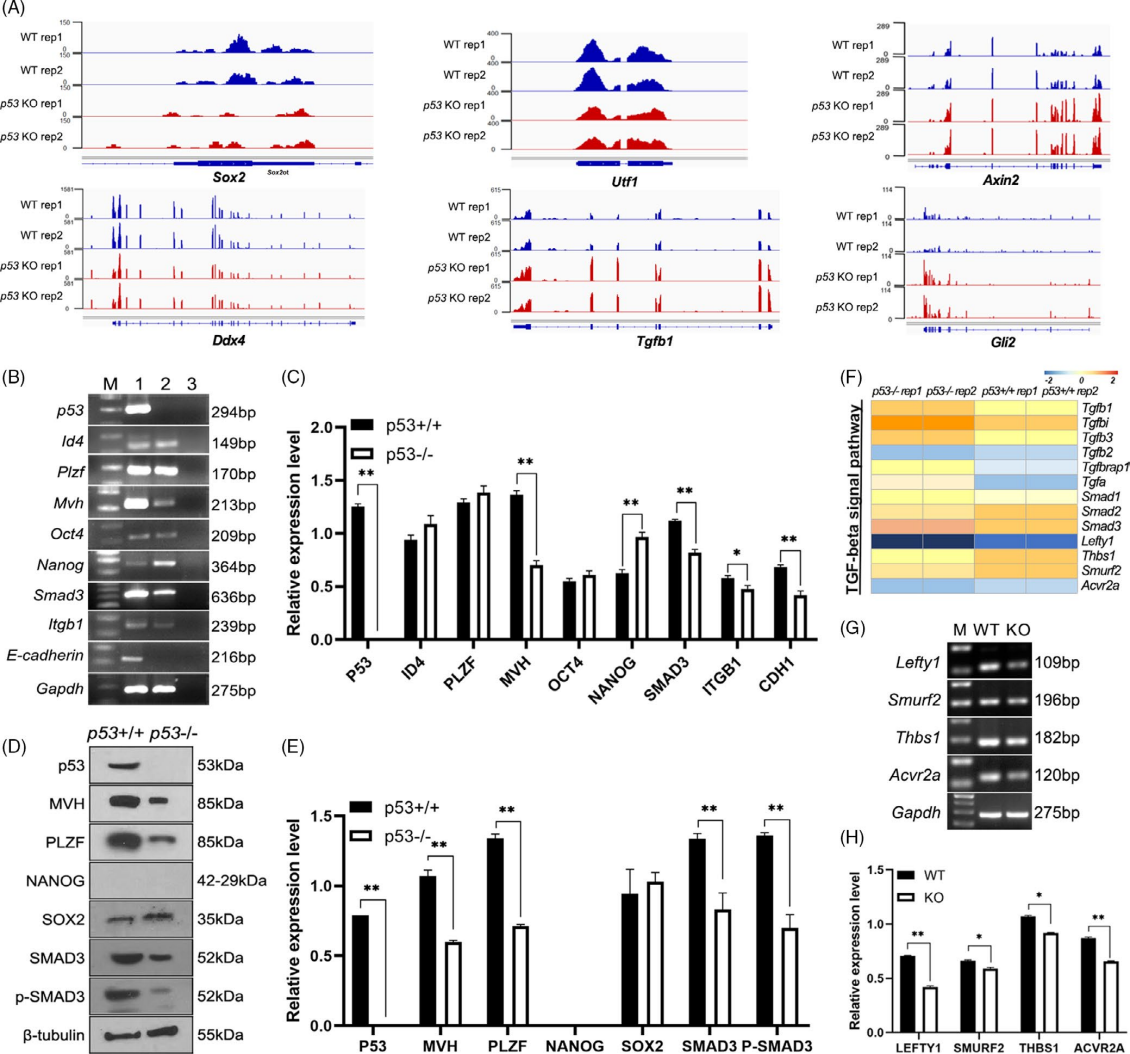
RNA‐seq and some genes related to pluripotency and reproductive system characteristics analysis of *p53* **^+/+^** and *p53* **^−/−^** SSCs (spermatogonial stem cells). Gene expression coverage maps for *Sox2*, *Utf1*, *Axin2*, *Ddx4*, *Tgfb1* and *Gli2* in *p53* ^+/+^ and *p53* ^−/−^ SSCs are exhibited (A). The expression levels of *p53*, *Id4*, *Plzf*, *Mvh*, *Oct4*, *Nanog*, *Smad3*, *Itgb1*, *E*‐*cadherin* and *Gapdh* were detected using reverse‐transcription‐polymerase chain reaction (RT‐PCR) in primary SSCs from *p53* ^+/+^ and *p53* ^−/−^ mice (M. marker, 1. *p53* ^+/+^ SSCs, 2. *p53* ^−/−^ SSCs, 3. H_2_O) (B), and were statistically analysed (*n* = 3) (C). The expression levels of p53, mouse vasa homologue (MVH), promyelocytic leukaemia zinc finger (PLZF), NANOG, SOX2, SMAD3 and p‐SMAD3 in primary SSCs from *p53* ^+/+^ and *p53* ^−/−^ mice were detected using Western blot (D) and were statistically analysed (*n* = 3) (E). Heatmap shows the expression levels of representative genes related to transforming growth factor‐ β (TGF‐β) signalling pathway in *p53* ^+/+^ and *p53* ^−/−^ SSCs (F). The expression levels of representative genes associated with TGF‐β signalling pathway were detected in *p53*+/+ (WT) and *p53*‐/‐ SSCs (KO) using RT‐PCR (G), and were statistically analysed (*n* = 3) (H). The data represent the means±SD (*, *p* < 0.05; **, *p* < 0.01)

Furthermore, we focused on six representative genes which were differentially expressed in *p53* ^+/+^ and *p53* ^−/−^ SSCs, including the pluripotency‐related genes *Sox2* and *Utf1*, germline marker gene *Ddx4* and signalling pathway‐related genes *Axin2*, *Gli2* and *Tgfb1* (Figure 5A). The expression levels of *Sox2* and *Utf1* were decreased in *p53* deficient SSCs, indicating that after deletion of *p53* gene, pluripotency was not immediately increased. However, both chromatin accessibility and expression levels of *Axin2*, *Gli2* and *Tgfb1*, the key genes of TGF‐β and Wnt signalling pathways, were activated, implying that p53 regulated their expression at chromosomal level. Interestingly, the transcription of germline marker gene *Ddx4* became inactive, and its chromatin was less accessible after *p53* deficiency (Table S6), suggesting the potential role of *p53* in the maintenance of *Ddx4* through regulating chromosomal state. Combined with the results of RNA‐seq that showed no significant increase in the expression levels of most pluripotent genes in *p53* ^−/−^ SSCs (Table S5), we further confirmed that *p53* deficient SSCs were still adopting germline identity, even if they already tended to transform into pluripotent state accompanied with decreased expression of some germline markers, which was probably related to TGF‐β and Wnt signalling pathways.

To verify the RNA‐seq results, we selected several genes associated with pluripotency, germline and TGF‐β signalling pathway to detect their expression at mRNA and protein levels. RT‐PCR results revealed that the expression levels of *Id4*, *Plzf* and *Oct4* were not remarkably changed, but the expression levels of some germline markers and spermatogonia markers including *Itgb1*, *E*‐*cadherin* and *Mvh* (*Ddx4*) were down‐regulated in *p53* ^−/−^ SSCs (Figure 5B,C), indicating that part of spermatogonia characteristics was decreased at mRNA level. However, the expression of *Smad3* was down‐regulated in *p53* deficient SSCs (Figure 5B,C), but RNA‐seq results showed that the expression of *Smad3* was not remarkably changed. To further verify these results, expression levels of these genes were determined at protein level. Both expression and phosphorylation levels of SMAD3 were down‐regulated by *p53* deficiency (Figure 5D,E), indicating declined function of the antiproliferative effector in *p53* deficient SSCs were decreased. And we noticed that the expression levels of *Cdk2* and *Cdk4*, two cell cycle kinases that regulate SMAD3 phosphorylation to inhibit SMAD3’s transcriptional activity and antiproliferative function,^47^ were about 1.39‐fold increased (Log2(fold value) = 0.477 and 0.471, respectively [Table S5]), and the expression of *Cdc25a*, the cell cycle gene inhibited by TGF‐β,^47^ was remarkably up‐regulated, confirming the down‐regulated expression and phosphorylation of SMAD3 after *p53* deficiency. Notably, the transformation of SSCs is a long‐term process, which needs more than 25 passages *in vitro*. Thus, we proposed that SMAD3 was not activated in the early stage of transformation. Similarly, we also noticed that the binding domain of ZFP57 (a key TF related to methylation regulation in pluripotency^48^) was more open (Figure 3F), but the chromatin region of *Zfp57* gene and transcriptional level of *Zfp57* were not increased in *p53* KO SSCs (Tables S4 and S5), indicating that *p53* loss led to the increased accessibility of ZFP57’s target genes, rather than affect *Zfp57* gene itself. The expression of *Nanog* was up‐regulated in *p53* deficient SSCs (Figure 5B,C), but in RNA‐seq data the expression of *Nanog* was not remarkably changed (Table S5). However, the expression values of *Nanog* detected by RNA‐seq were 0.087 and 0, respectively, indicating that the expression level was too low to be detected using RNA‐seq. Moreover, NANOG signal was detected neither in *p53* **^+/+^** nor in *p53* **^−/−^** SSCs using Western blot (Figure 5D,E). This extremely low expression level of *Nanog* may explain the inconsistency between molecular assays and RNA‐seq. Since the sensitivity of RT‐PCR and Western blot was higher than that of RNA‐seq, we confirmed that the expression of *Nanog* was up‐regulated at mRNA level after *p53* loss, and its chromosomal accessibility was increased, as well.

Subsequently, the expression changes of genes in TGF‐β signalling pathway detected in RNA‐seq were analysed and exhibited in a heatmap (Figure 5F), and the expression levels of these genes were verified using RT‐PCR (Figure 5G,H). Compared with *p53* ^+/+^ SSCs, the expression levels of all selected genes: *Lefty1*, *Smurf2*, *Thbs1* and *Acvr2a* were significantly down‐regulated in *p53* ^−/−^ SSCs. As upstream repressive genes of the TGF‐β family, *Lefty1*, *Smurf2*, *Thbs1* and *Acvr2a* bind to various TGF‐β receptors to recruit and activate SMAD family transcription factors, to regulate gene expression. These results further confirmed that TGF‐β signalling pathway was activated in *p53* deficient SSCs, and we therefore proposed that TGF‐β signalling pathway was pivotal for SSCs spontaneous transformation.

### 3.6 Pivotal genes associated with pluripotent signal network in differential accessible regions and potential mechanism

Next, we analysed the expression levels of TFs whose binding domains were more accessible after *p53* deficiency (Table 1). Although the binding regions of these 29 TFs (Figure 3F) were more accessible after *p53* deletion, the transcriptional activity of TFs was not up‐regulated (Table S5), indicating that *p53* is associated with the chromosomal structure of these TFs’ target genes, rather than regulating the expression levels of these TFs. Moreover, the expression levels of pluripotent genes, such as *Sox2* and *Nanog*, were not up‐regulated, but the accessibility of their binding domains was increased, indicating that their chromosomal states were affected by *p53* deficiency. On the other hand, although the expression of these pluripotency‐associated TFs, such as SMAD3, was not increased either, it should be noted that this did not mean that the expression levels of SMAD3’s target genes were not changed, since the increased chromatin openness of target genes normally enhances the binding efficiency of TF to regulate gene expression.^49^ Therefore, the target genes of SMAD3 were possibly affected by *p53* deficiency, for example, *Sox2* was predicted as a putative target of SMAD3, and the expression of *Sox2* was positively related to SMAD3 expression (Table 2), indicating a direct regulatory effect of SMAD3 on *Sox2* in SSCs.

**TABLE 1:** The expression changes of transcription factors that recognize the accessibility increased domains after *p53* deficiency in spermatogonial stem cells (SSCs)

gene_ID	locus	sample_1	sample_2	value_1(Sample_1)	value_2 (Sample_2)	log2 (fold_change)	Relative expression (*p53* KO vs WT)
*Ctcf*	chr8:105636346–105682924	*p53* KO	WT	17.851	23.3467	−0.38722	\
*E2f4*	chr8:105297662–105305370	*p53* KO	WT	28.003	19.8986	0.492913	\
*Smad3*	chr9:63646764–63757994	p53 KO	WT	27.0548	21.1877	0.352657	\
*Smad4*	chr18:73639012–73703741	p53 KO	WT	36.8651	45.3384	−0.29848	\
*Pou5f1*	chr17:35506031–35510777	p53 KO	WT	27.1936	34.8058	−0.35606	\
*Dmrt3*	chr19:25610536–25623921	p53 KO	WT	9.87694	8.14039	0.278966	\
*Stat1*	chr1:52119437–52233232	p53 KO	WT	10.9315	9.66132	0.178198	\
*Sox2*	chr3:34560380–34677993	*p53* KO	WT	0.687556	1.95592	−1.5083	Down‐regulated
*Elk4*	chr1:132007604–132068062	*p53* KO	WT	4.46601	7.87563	−0.81841	Down‐regulated
*Mybl2*	chr2:163054634–163084732	*p53* KO	WT	20.8014	10.9112	0.930868	Up‐regulated

Note

According to the results of ATAC‐seq, among the transcription factors corresponding to the open transcription factor binding domain, we selected the transcription factors that may be related to pluripotency transformation, including *Ctcf*, *E2f4*, *Smad3*, *Smad4*, *Stat1*, *Sox2*, *Pou5f1*, *Dmrt3*and *Mybl2*. *Sox2* and *Elk4* expression was down‐regulated, while *Mybl2* expression was significantly increased (Log2FC>0.585 means expression level is remarkably increased after *p53* KO,Log2FC< −0.585 means decreased).

**TABLE 2:** Prediction of the binding sites of SMAD3 in some pluripotency‐associated genes

gene_ID	locus	sample_1	sample_2	value_1(Sample_1)	value_2 (Sample_2)	log2 (fold_change)	Relative expression (*p53* KO vs WT)
*Foxo1*	chr3:52268336–52350109	*p53* KO	WT	34.7167	49.0257	−0.49791	\
*Mycn*	chr12:12936092–12941996	*p53* KO	WT	25.9709	33.9265	−0.38552	\
*Nanog*	chr6:122707488–122714633	*p53* KO	WT	0.087124	0	‐‐	\
*Sall4*	chr2:168748331–168767201	*p53* KO	WT	37.6655	41.1992	−0.12937	\
*Nodal*	chr10:61417971–61425337	*p53* KO	WT	0.148573	0.075261	0.981201	\
*Axin2*	chr11:108920348–108950781	*p53* KO	WT	7.96963	4.57413	0.801016	Up‐regulated
*Tgfb1*	chr7:25687001–25704996	*p53* KO	WT	17.7919	9.58317	0.892643	Up‐regulated
*Tgfb3*	chr12:86056742–86079041	*p53* KO	WT	17.8388	10.4755	0.767994	Up‐regulated
*Cdh1*	chr8:106603209–106670248	*p53* KO	WT	11.5823	21.3651	−0.88334	Down‐regulated
*Itgb1*	chr8:128685653–128733579	*p53* KO	WT	61.5212	110.308	−0.84239	Down‐regulated
*Sox2*	chr3:34560380–34677993	*p53* KO	WT	0.687556	1.95592	−1.5083	Down‐regulated

Note

Log2FC>0.585 means expression level is remarkably increased after *p53* KO,Log2FC< −0.585 means decreased.

Log2FC>0.585 means expression level is remarkably increased after *p53* KO,Log2FC< −0.585 means decreased.

Subsequently, we analysed whether SMAD3 could bind to some pluripotency‐associated genes and found SMAD3’s potential binding sites in *Foxo1*, *Axin2*, *Cdh1*, *Itgb1*, *Mycn*, *Nanog*, *Nodal*, *Sall4*, *Sox2*, *Tgfb1* and *Tgfb3* genes. Consistently, expression levels of *Axin2*, *Tgfb1* and *Tgfb3* were up‐regulated (Table 2), suggesting the direct binding and regulatory effect on these genes. Above conclusions showed that SMAD3 was not activated in SSCs. As a potential target of SMAD3, the expression of *Nanog* was extremely low, which was not detectable at protein level in wild‐type and *p53* knockout SSCs (Figures 2D and 5C,D). It was not sure whether the low expression of *Nanog* was associated with transcriptional level of *Smad3* in SSCs. Moreover, we analysed the binding sites of SMAD4, which cooperates with SMAD3 for DNA binding. The potential binding sites were predicted in *Cdh1*, *Itgb1*, *Mycn*, *Nanog*, *Nodal*, *Sall4*, *Foxo1*, *Tgfb1* and *Tgfb3* genes (Table 3), which were very close to those of SMAD3, and the only difference was that the binding sites were not found in *Sox2 and Axin2*. These results further suggest that SMAD3 may cooperate with SMAD4 to bind to and regulate the above‐mentioned pluripotency‐related genes in *p53* deficient SSCs, and SMAD3 probably directly binds to and regulates *Sox2* and *Axin2* genes.

**TABLE 3:** Prediction of the binding sites of SMAD4 in some pluripotency‐associated genes

gene	locus	sample_1	sample_2	value_1	value_2	log2.fold_change	Relative expression (*p53* KO vs WT)
*Itgb1*	chr8:128685653–128733579	treat	ctrl	OK	61.5212	110.308	\
*Mycn*	chr12:12936092–12941996	treat	ctrl	25.9709	33.9265	−0.38552	\
*Foxo1*	chr3:52268336–52350109	treat	ctrl	34.7167	49.0257	−0.49791	\
*Sall4*	chr2:168748331–168767201	*p53* KO	WT	37.6655	41.1992	−0.12937	\
*Nanog*	chr6:122707488–122714633	*p53* KO	WT	0.087124	0	‐‐	\
*Cdh1*	chr8:106603209–106670248	treat	ctrl	11.5823	21.3651	−0.88334	Down‐regulated
*Nodal*	chr10:61417971–61425337	*p53* KO	WT	0.148573	0.075261	0.981201	Up‐regulated
*Tgfb1*	chr7:25687001–25704996	treat	ctrl	17.7919	9.58317	0.892643	Up‐regulated
*Tgfb3*	chr12:86056742–86079041	treat	ctrl	17.8388	10.4755	0.767994	Up‐regulated

Note

Log2FC>0.585 means expression level is remarkably increased after *p53* KO,Log2FC< −0.585 means decreased.

### 3.7 SMAD3/SMAD4 regulates the pluripotent signalling network in SSCs

**FIGURE 6:**
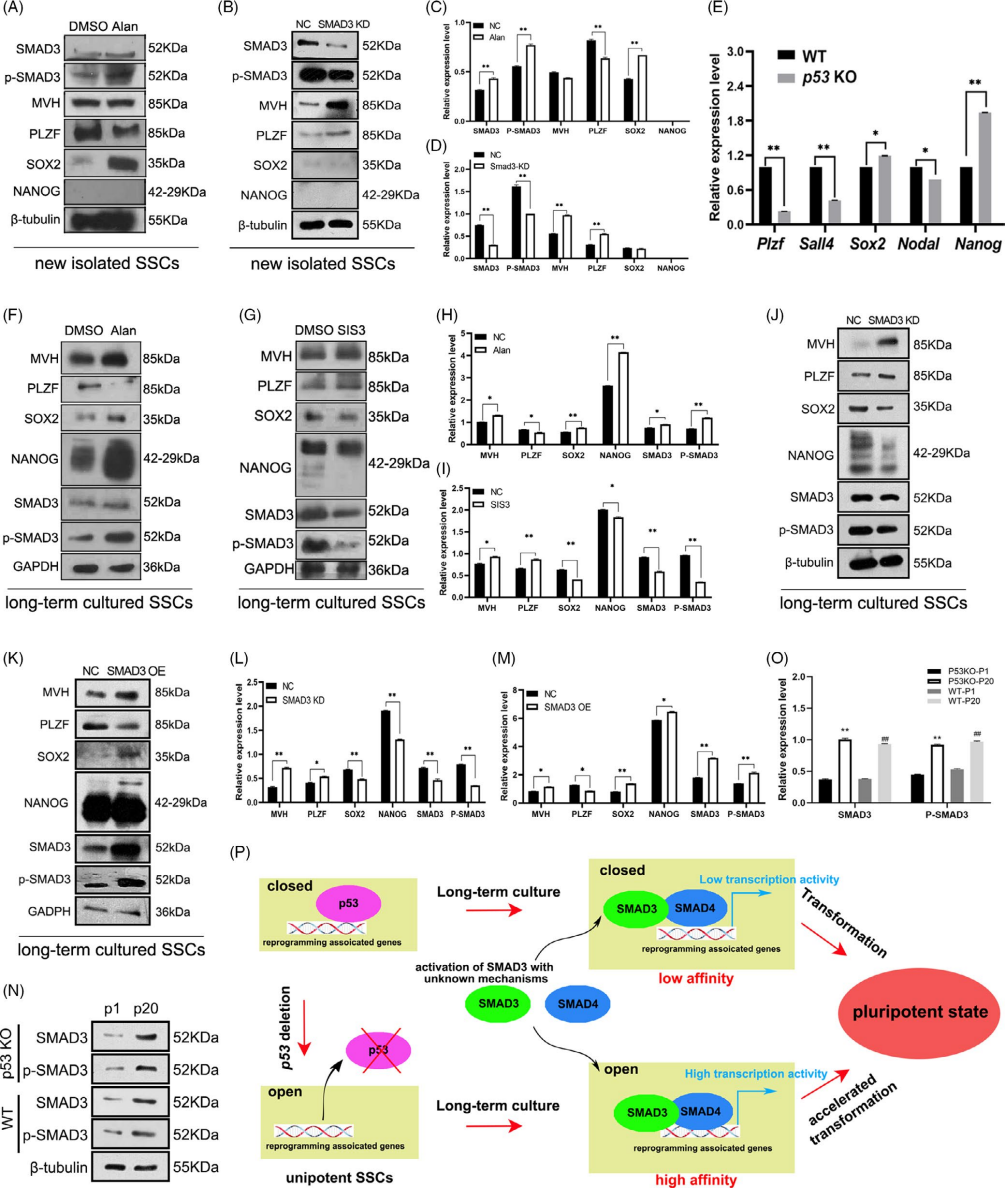
SMAD3 plays an important role in regulating the pluripotent signalling network in spermatogonial stem cells (SSCs). The expression levels of mouse vasa homologue (MVH), NANOG, SOX2, SMAD3 and p‐SMAD3 in primary SSCs treated with alantolactone (10 μM, 6h) (A) or scrambled/*Smad3* siRNA (B) were detected using Western blot, and the results of Alan‐treated (C) or small interfering RNA (siRNA)‐treated groups (D) were statistically analysed (*n* = 3). *p53* ^+/+^ and *p53* ^−/−^ SSCs cultured *in vitro* for 20 passages were harvested to detect the relative expression levels of germline markers *Plzf*, *Mvh*, and pluripotent markers *Sox2*, *Nodal* and *Nanog* (*n* = 3) (E). SSCs cultured *in vitro* for 20 passages were incubated with alantolactone (F), SMAD3 inhibitor SIS3 (G), scrambled/Smad3 siRNA (J) or pcDNA3‐*Smad3* (K) for 48 h, and expression levels of MVH, promyelocytic leukaemia zinc finger (PLZF), SOX2, NANOG, SMAD3 and glyceraldehyde 3‐phosphate dehydrogenase **(**GAPDH), and phosphorylation level of SMAD3 were detected, and these results were statistically analysed (H. Alan‐treated long‐term SSCs, I. SIS3‐treated long‐term SSCs, L. *Smad3* siRNA transfected long‐term SSCs, M. pcDNA3‐*Smad3* transfected long‐term SSCs. *n* = 3). The *p53* KO and wild‐type (WT) SSCs of passage 1 and passage 20 were harvested to determine the expression and phosphorylation of SMAD3 using Western blot (N), and the results were statistically analysed (*n* = 3) (O). Illustration of the potential regulatory network of *p53* in mediating pluripotency transformation of SSCs (P). The data represent the means±SD (*, *p* < 0.05; **, *p* < 0.01)

Above observations demonstrated the potential connection of SMAD3 and many pluripotency‐associated genes. However, it is unexpected that the expression level of SMAD3 in newly isolated *p53* ^−/−^ SSCs was lower than that in *p53* ^+/+^ SSCs (Figure 5B–E), indicating that loss of *p53* could not activate expression or phosphorylation of SMAD3. Thus, we wonder whether activation of SMAD3 could regulate these pluripotent genes in SSCs. First, newly isolated SSCs were treated with SMAD3 activator alantolactone to enhance the SMAD3 activity, leading to down‐regulated expression of PLZF and up‐regulated expression of SOX2 in alantolactone‐treated SSCs (Figure 6A,C). Combined with the bioinformatic prediction that SMAD3 could potentially bind to *Sox2* gene, we conjectured that SMAD3 positively regulated *Sox2* expression in SSCs. However, NANOG was still not detected at protein level (Figure 6A,C), indicating that the short‐term activation of SMAD3 in newly isolated SSCs was insufficient to induce SSCs transformation, and we further confirmed that transformation of SSCs into pluripotency is a long‐term process which probably takes several steps. To eliminate the off‐targets effect of small molecule, expression of *Smad3* was disturbed in newly isolated SSCs using small interfering RNA (siRNA). Consistently, expression levels of MVH and PLZF were up‐regulated, while the signals of SOX2 and NANOG were too weak to be observed (Figure 6B,D).

Subsequently, we cultured *p53* ^+/+^ and *p53* ^−/−^ SSCs for around 20 passages *in vitro*, to detect the expression change of germline and pluripotent genes. Expression of *Sox2* and *Nanog* was detected (Figure 6E), confirming that after long‐term culture, both *p53* ^+/+^ and *p53* ^−/−^ SSCs gradually transformed, at least could be observed at mRNA level. Meanwhile, expression levels of germline marker were decreased and pluripotent markers were significantly increased in *p53* ^−/−^ SSCs (Figure 6E), confirming that transformation efficiency was higher in *p53* deficient SSCs. Therefore, we proposed that these long‐term cultured *p53* ^+/+^ and *p53* ^−/−^ SSCs partially possessed some of the characteristics of pluripotent stem cells at molecular levels, and selected SSCs around 20 passages to study the regulatory role of SMAD3 in SSCs transformation. The dosage effect and treatment time of SMAD3 activator alantolactone or SMAD3 inhibitor SIS3 were tested in these long‐term cultured SSCs (Figure S3). Alantolactone treatment increased expression levels of SOX2, SMAD3 and NANOG, and enhanced the phosphorylation level of SMAD3 (Figure 6F,H). Consistently, SIS3 remarkably inhibited the expression of SOX2, NANOG, SMAD3, and inhibited SMAD3 phosphorylation (Figure 6G,I). And the expression of SSCs marker PLZF was negatively regulated by SMAD3 (Figure 6F–I), implying that activation of SMAD3 promoted SSCs transformation and led to the loss of SSCs characteristics. Likewise, expression of *Smad3* was disturbed in SSCs around 20 passages using siRNA to eliminate the off‐target effects of small molecules, and identical effect on the expression of germline and pluripotent markers was observed (Figure 6J,L). As expected, transfection of SMAD3 expression plasmid in long‐term SSCs enhanced the expression levels of SOX2 and NANOG (Figure 6K,M), confirming the regulatory role of SMAD3 in pluripotency in the later stage of SSCs transformation. Finally, we noticed that the expression level and phosphorylation level of SMAD3 were remarkably up‐regulated after long‐term culture in *p53* KO and wild‐type SSCs (Figure 6N,O). Combined with the results that the expression level of SMAD3 in newly isolated *p53* ^−/−^ SSCs was lower than that of wild‐type control (Figure 5B–E), we proposed that SMAD3 activation occurred in the middle or late stage of transformation, to further promote NANOG expression.

Based on the reported conclusions and results of this study, we proposed a potential model of *p53* in mediating pluripotency transformation of SSCs (Figure 6P). As a tumour suppressor, p53 inhibits many target genes and maintains the stability of chromosomes. Deletion of *p53* increases accessibility of many domains in chromatin, and TFs detected in the ATAC‐seq assay (such as CTCF, OCT4:SOX2 complex, E2F4, SMAD3 and SMAD4) can bind to these opened regions to regulate their expression more efficiently. Some of them are reprogramming‐associated genes, including Yamanaka factors, *c*‐*Myc*, *Sox2*, and they were identified as the putative targets of SMAD3, SMAD4 and E2F4 in this study. Additionally, SSCs endogenously express *Oct4* (Figure 2D). Loss of *p53* provides many, if not all, essential conditions for pluripotency transformation, and activation of *Nanog*, the core gene of pluripotency, is more efficient during *in vitro* culture. However, expression and phosphorylation of SMAD3 are not up‐regulated after *p53* deletion, and *p53* deficient SSCs still need long‐term culture to transform into pluripotent fate, indicating a key event may happen during this process, which is the prerequisite to activate SMAD3 and induce SSCs transformation. This is similar to “two‐hit hypothesis” in tumorigenesis,^50^ that *p53* deficient SSCs fail to maintain their fate when a stimulus appears. In the future research, we will screen this unknown event of SSCs transformation to pluripotent fate.

## 4 DISCUSSION

The relationship between *p53* and pluripotency is complex. Here, we observed that *p53* loss led to open chromatin state of *Nanog*, and increased expression of *Nanog* mRNA. However, *p53* deficiency was insufficient to drive SSCs transformation, since most of the pluripotent genes were not up‐regulated (especially NANOG protein was not detectable), and germline characteristics still remained. Increased expression of NANOG at protein level was detected only in long‐term culture SSCs, which was further enhanced by SMAD3 activator or overexpression. Considering that SSCs transformation composed of several steps, we proposed that SMAD3 was not activated in the early stage, and the underlying mechanism in the whole transformation process was more complicated than our expectation. Besides the role of inhibiting *Nanog* expression in pluripotent transformation, evidence showed that *p53* deficiency promoted reprogramming by avoiding cell arrest or apoptosis.^18^ *p53* also regulates pluripotent genes such as Leukaemia Inhibitory Factor (LIF) and some long non‐coding RNAs (lncRNAs) to maintain the pluripotency of ESC.^18^ On the other hand, inhibition of p53 activity is the key to maintaining the versatility of ESCs under stress‐free condition.^51^ The results of ATAC‐seq showed that the binding domains of OCT4:SOX2, E2F4, SMAD3 and other transcription factors were more accessible in *p53* deficient SSCs, and the key adhesion signal genes, such as *Itgb1* and *E*‐*cadherin*, were down‐regulated. This observation implied that p53 played a role for SSCs maintenance by inhibiting the accessibility of chromosomal regions by recognizing pluripotent transcription factors. Based on these conclusions and our observations, we propose that p53 is more likely to be responsible for maintaining the original cell fate under the stimulation of inducing factors. If the change of cell fate is irreversible, it will trigger cell stagnation or death. In this case, loss of *p53* is a key step for cell differentiation or reprogramming.

It is worth noting that the activity of *Oct4* is essential during the embryo development of sperm‐egg binding.^52^ Our observations are consistent with reported evidence^53^, ^54^ that regulation of *Oct4* expression and stemness is closely associated. In germline, *Oct4* expression is maintained at a low level, while after fertilization or reprogramming, the expression of *Oct4* is activated and cells obtain pluripotency.

Although the accessibility of many domains is increased in *p53* deficient SSCs, neither the expression levels of TFs we identified nor the expression levels of pluripotent genes recognized by these TFs were significantly up‐regulated in *p53* KO SSCs. This suggests that *p53* deleted SSCs retain the characteristics of germ cells and have not obtained pluripotency, yet, and that is why SSCs need further culture *in vitro* to transform into pluripotent state. Typically, in primary SSCs, alantolactone is able to stimulate SOX2 expression, indicating that SMAD3 activation is able to drive pluripotency transformation at the early stage, even though NANOG was not detectable at protein level (Figure 6A). When SSCs around 20 passages already expressed NANOG, the expression level of NANOG was positively related to SMAD3 (Figure 6). These observations also suggest that SMAD3 promotes pluripotency transformation throughout SSCs culture, but in the beginning, there is no inducing factor to activate SMAD3 expression. Thus, SSCs transformation takes several steps, which may be divided by the expression of NANOG protein as a key event, and the expression of *Nanog* gene is regulated by SMAD3 throughout the process. Moreover, SMAD3 probably plays different roles in each step.

A study revealed that phosphorylated SMAD2 or SMAD3 could bind to the *Nanog* proximal promoter region to regulate *Nanog* expression in human ESCs and in mouse epiblast stem cells (EpiSCs).^55^ We also predicted *Nanog* as a direct target of SMAD3 and SMAD4 according to the bioinformatics analysis (Tables 2 and 3). Thus, we proposed that SMAD3 positively regulates the expression of *Nanog*, to regulate the pluripotency transformation of SSCs. However, further molecular evidence is required to support this hypothesis. This also suggested that the regulation of stem cells’ fate may be related to multiple signal networks, and the regulatory effect of a single transcription factor is limited.

Although the accessibility of SMAD3’s binding domains was increased in the newly isolated *p53* deficient SSCs (Figure 3F), it is not clear why the expression level and phosphorylation level of SMAD3 were declined in these cells (Figure 5D). Considering that alantolactone treatment enhanced the expression of pluripotent genes and attenuated the expression of germline genes both in newly isolated SSCs and in long‐term culture SSCs, we propose that there are probably some endogenous mechanisms to prevent SSCs transformation, and an essential factor/step is required to activate SMAD3 expression in SSCs, which is the prerequisite of SSCs transformation. TGF‐β signalling pathway is probably involved in this unknown event. However, further research is needed to verify this hypothesis.

In addition, the relationship between *p53* and E2F4 is also very important. We found that in *p53* deficient SSCs, the openness of E2F4’s binding domain was also increased. E2F4 can form a complex with SMAD3, which enters the nucleus under the stimulation of TGF‐β and combines with SMAD4 to regulate the expression of *c*‐*Myc*.^56^ Therefore, SMAD3 and SMAD4 may activate *c*‐*Myc* and *Nanog* to promote SSCs transformation by interacting with E2F4. This hypothesis needs to be further investigated, as well.

Shinohara's team revealed that SSCs transformation was associated with *p53* and *Dnmt1*. Deficiency of *p53* combining with epigenetic changes jointly affected the genomic stability and expression profile of SSCs, which were also involved in the cooperation with OCT4: SOX2. Here, we noticed that the expression levels of *Dnmt1* and *Dmrt1* were not significantly changed, indicating that methylation modification has not occurred, yet. And we observed that the expression levels of SMAD3/4’s target genes *Itgb1* and *E*‐*cadherin* decreased in *p53* deficient SSCs, indicating the reduced cell adhesion. Considering that this event can enhance the undifferentiated state of pluripotent stem cells,^57^ we believe that the altered cell surface interaction is also important for SSCs transformation.

A study compared the expression profiles of SSCs and reprogrammed pluripotent SSCs using RNA‐seq, and predicted some potential transcription factors associated with three pluripotency‐related processes including cell proliferation, stem cell maintenance and epigenetic regulation.^58^ Totally, 15 TFs were predicted as two groups, 4 of them (OCT4, CUX1, ZFP143, E2F4) were associated with the early stage of reprogramming and 11 regulated pluripotency‐related processes at the late stage, based on bioinformatics analysis. Here, the chromatin accessibility changes demonstrated using ATAC‐seq provided direct biological evidence for key TFs associated with SSCs transformation. Consistently, OCT4 and E2F4 were also identified in our system. The TFs identified in two studies were not identical, since Jeong et al. collected data from normal SSCs and transformed SSCs, while we selected newly isolated *p53* ^+/+^ and *p53* ^−/−^ SSCs for analysis.

## 5 CONCLUSION

This study explored the impact of *p53* deletion on the fate of SSCs and the underlying molecular mechanism. Due to the complexity of p53’s function and regulatory network, we screened regions with increased accessibility in the whole chromosome, to analyse the opening degree of key genes related to pluripotency induced by *p53* deficiency, and revealed the molecular mechanism of SSCs transformation. This study is helpful to understand the role and molecular mechanism of *p53* in maintaining the genome stability and cell fate, which is conducive to revealing the connection of stemness transition at chromatin level, and will provide theoretic reference for ageing, tumour biology or clinical research.

## CONFLICT OF INTEREST

The authors have no potential conflicts of interest.

## AUTHOR CONTRIBUTIONS

Sitong Liu: Collection and assembly of data, data analysis and interpretation, manuscript writing. Rui Wei: Collection and assembly of data, data analysis and interpretation, manuscript writing. Hongyang Liu: Collection and assembly of data. Ruiqi Liu: Collection and assembly of data. Pengxiao Li: Data analysis and interpretation. Xiaoyu Zhang: Data analysis and interpretation. Xiaodong Zhao, Data analysis. Xiaomeng Li: Data analysis, financial support. Yang Yang: Data analysis. Xueqi Fu: Conception and design, financial support. Kang Zou: Conception and design, financial support, manuscript writing, final approval of manuscript. All of the authors in the authorship list agreed to publication of this study.

## Supporting information

Fig S1

Fig S2

Fig S3

Table S1

Table S2

Table S3

Table S4

Table S5

Table S6

Table S7

## Data Availability

The data that support the findings of this study are available in the method part and supplemental materials.
