# Supplementary figures and images for: Analysis of chromatin accessibility in p53 deficient spermatogonial stem cells for high frequency transformation into pluripotent state
Source: Cell Prolif. 2022 Feb 4;55(3):e13195. doi: 10.1111/cpr.13195 (PMC8891552; doi:10.1111/cpr.13195)

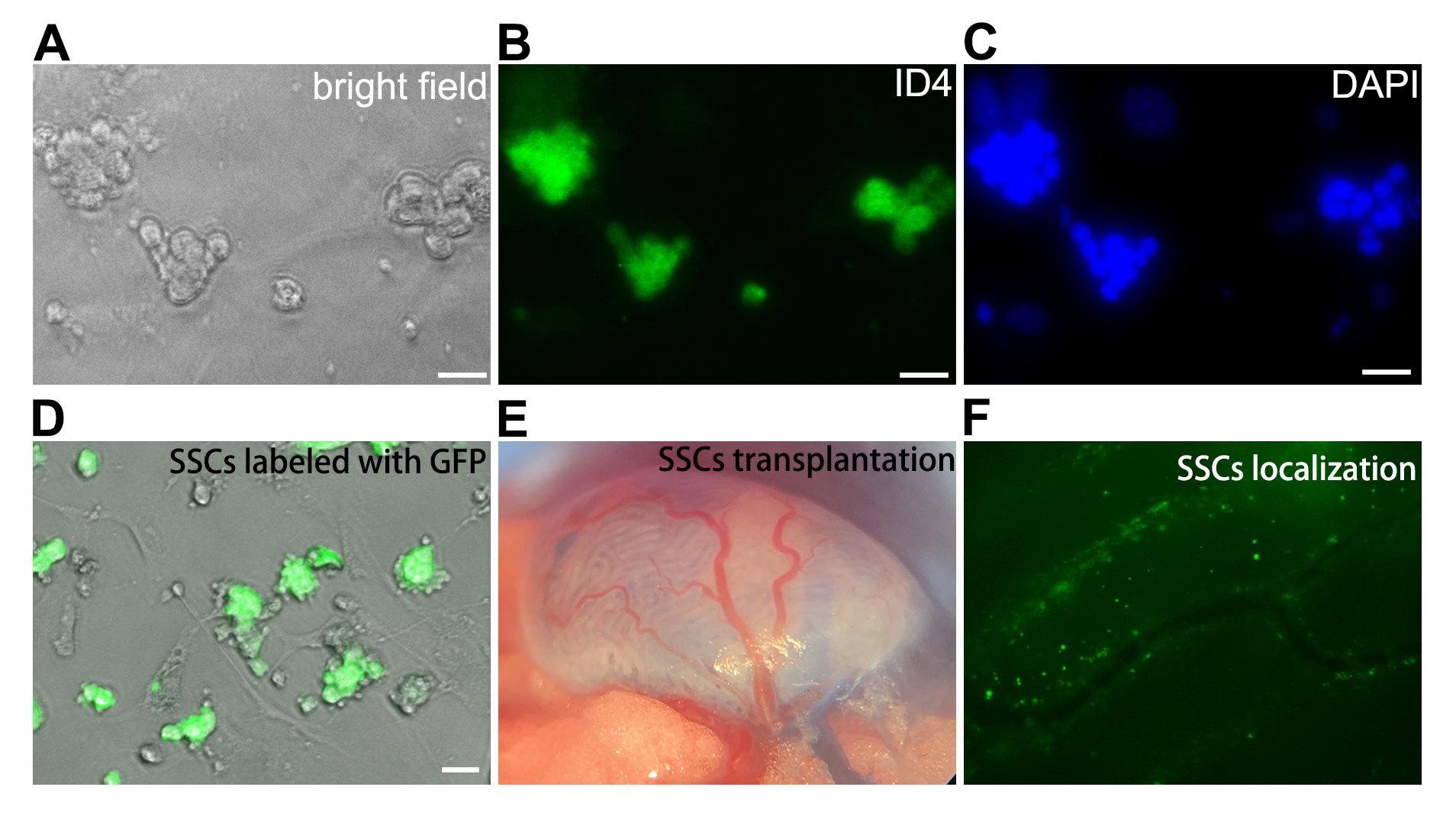

Supplement: Supplementary file 1 — Fig S1 [file CPR-55-e13195-s008.tif]

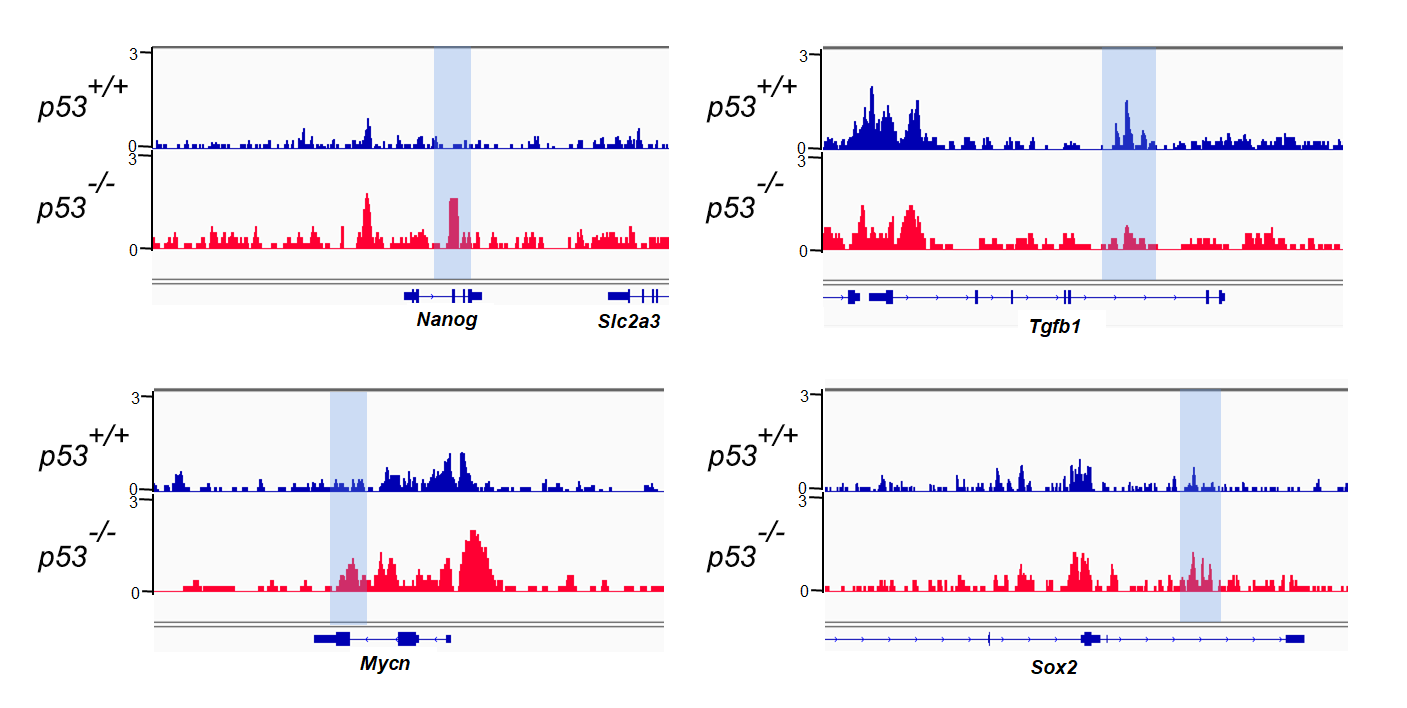

Supplement: Supplementary file 2 — Fig S2 [file CPR-55-e13195-s005.tif]

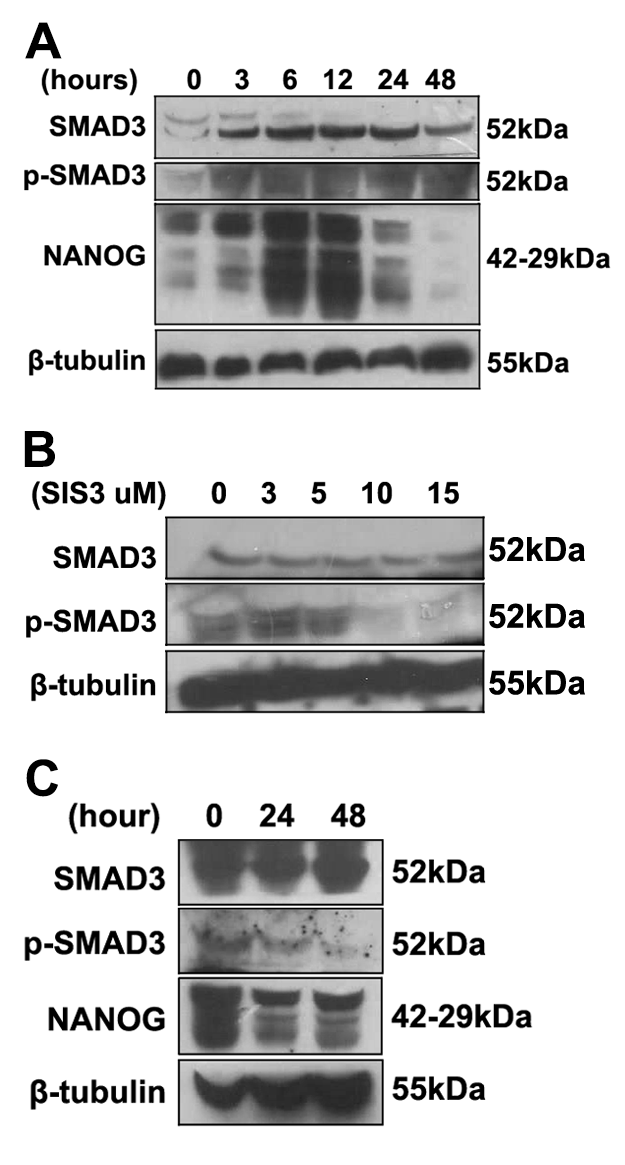

Supplement: Supplementary file 3 — Fig S3 [file CPR-55-e13195-s007.tif]
