# Supplementary material for: Analysis of chromatin accessibility in p53 deficient spermatogonial stem cells for high frequency transformation into pluripotent state
Source: Cell Prolif. 2022 Feb 4;55(3):e13195. doi: 10.1111/cpr.13195 (PMC8891552; doi:10.1111/cpr.13195)
Supplement: Supplementary file 4 — Table S1 [file CPR-55-e13195-s006.docx]

**Table S1. The components of Shinohara’s germline stem cells medium and ESC medium.**

| **REAGENT** | **SOURCE** | **IDENTIFIER** | **Modified Shinohara’s germline stem cells medium** | **ESC medium** |
| --- | --- | --- | --- | --- |
| IMDM | Gibco | 12200-036 | ✓ | - |
| DMEM | Gibco | 12100-046 | - | ✓ |
| Pyruvic acid | Sigma | P2256 | 30 μg/ml | 30 μg/ml |
| D-(+)-glucose | Sigma | G7021 | 6 mg/ml | - |
| DL-Lactic Acid | Sigma | L4263 | 1 μl/ml | - |
| Bovine albumin (BSA) | MP Biomedicals | b810661 | 5 mg/ml | - |
| L-Glutamine | Sigma | G7513 | 2 mM | 2 mM |
| 2-Mercaptoethanol | Sigma | M3158 | 5×10^-5^ M | 5×10^-5^ M |
| MEM Vitamin Solution | Invitrogen | 11120-052 | 1× | - |
| Non-Essential Amino Acids | Invitrogen | 11140-050 | 1× | 1× |
| Ascorbic Acid | Sigma | A4544 | 10^-4^ M | - |
| d-Biotin | Sigma | B4501 | 10 μg/ml | - |
| β-Estradiol | Sigma | E2758 | 30 ng/ml | - |
| FBS | Gibco | 1600-044 | 1% | 15% |
| Knockout Serum Replacement | Invitrogen | 10828-028 | 50 μl/ml | - |
| N2 | Thermo Fisher Scientific | 17502-048 | 1× | - |
| Human FGF2 | Peprotech | 100-18b | 10 ng/ml | - |
| Rat GDNF | Peprotech | 450-51 | 15 ng/ml | - |
| Human LIF | Peprotech | 300-05 | - | 10 ng /ml |
