# Supplementary material for: Analysis of chromatin accessibility in p53 deficient spermatogonial stem cells for high frequency transformation into pluripotent state
Source: Cell Prolif. 2022 Feb 4;55(3):e13195. doi: 10.1111/cpr.13195 (PMC8891552; doi:10.1111/cpr.13195)
Supplement: Supplementary file 5 — Table S2 [file CPR-55-e13195-s001.docx]

**Table S2.** **The information of antibodies**

| **Antibodies** | **SOURCE** | **IDENTIFIER** |
| --- | --- | --- |
| Polyclonal rabbit anti-mouse PLZF | Santa Cruz Biotechnology | sc-22839 |
| Monoclonal mouse anti-mouse Tubulin | Absin Bioscience | Abs830032ss |
| Monoclonal mouse anti-mouse P53 | Santa Cruz Biotechnology | sc-393031 |
| Monoclonal mouse anti-mouse Oct3/4 | Santa Cruz Biotechnology | sc-5279 |
| Monoclonal rabbit anti-mouse MVH | Cell Signaling Technology | 8761 |
| Monoclonal rabbit anti-mouse Nanog | Cell Signaling Technology | 8822 |
| Monoclonal rabbit anti-mouse Sox2 | Cell Signaling Technology | 23064 |
| Monoclonal rabbit anti-mouse Smad3 | Cell Signaling Technology | 9523 |
| Monoclonal rabbit anti-mouse p-Smad3 (ser423/425) | Cell Signaling Technology | 9520 |
| Goat anti rabbit IgG – HRP conjugated | Santa Cruz Biotechnology | sc-2005 |
| Goat anti mouse IgG – HRP conjugated | Santa Cruz Biotechnology | sc-2004 |
| Goat anti rabbit IgG – red | Santa Cruz Biotechnology | sc-2091 |
| Goat anti mouse IgG – red | Santa Cruz Biotechnology | sc-2092 |
