# Supplementary material for: Analysis of chromatin accessibility in p53 deficient spermatogonial stem cells for high frequency transformation into pluripotent state
Source: Cell Prolif. 2022 Feb 4;55(3):e13195. doi: 10.1111/cpr.13195 (PMC8891552; doi:10.1111/cpr.13195)
Supplement: Supplementary file 6 — Table S3 [file CPR-55-e13195-s010.docx]

**Table S3. Information of primers**

| Gene | Accession  number | Product  Size(bp) | Primer Sequence (5’-3’) | Region  Amplified |
| --- | --- | --- | --- | --- |
| *p53* | NM_001127233 | 294 | F: CACGTACTCTCCTCCCCTCAA | 520-813 |
|  |  |  | R: GGCTCATAAGGTACCACCACG |  |
| *Id4* | NM_031166.3 | 149 | F: GAGACTCACCCTGCTTTGCT | 707-855 |
|  |  |  | R: ATGCTGTCACCCTGCTTGTT |  |
| *Plzf* | NM_001033324.3 | 170 | F: ACCAGTGTACCATCTGCACG | 2132-2301 |
|  |  |  | R: CTGCTCTACCATGTGTTGGG |  |
| *Mvh* | NM_001145885.1 | 213 | F: GGAAACCAGCAGCAAGTGAT | 607-819 |
|  |  |  | R: TGGAGTCCTCATCCTCTGG |  |
| *Itgβ1* | NM_010578.2 | 239 | F: GTCTGTTTGCAATATGGGGG | 3527-3765 |
|  |  |  | R: GCACTGTCAAAATGAAAAGGC |  |
| *c-Kit* | NM_001122733.1 | 222 | F: TCATCGAGTGTGATGGGAAA | 511-732 |
|  |  |  | R: GTGACTTGTTTCAGGCACA |  |
| *Gapdh* | NM_001289726.1 | 133 | F: CCTGGAGAAACCTGCCAAGTATG | 830-962 |
|  |  |  | R: AGAGTGGGAGTTGCTGTTGAAGTC |  |
| *Gfra1* | NM_001285457.2 | 208 | F: AGAAGCAGTTTCACCCAG | 1931-2138 |
|  |  |  | R: ATCATCACCACCACCATC |  |
| *Integrin-a6* | NM_001277970.1 | 269 | F: GAGGAATATTCCAAACTGAACTAC | 3087-3355 |
|  |  |  | R: GGAATGCTGTCATCGTACCTAGAG |  |
| *E-cad* | NM_009864.3 | 216 | F: ACCGATTCAAGAAGCTGGC | 2751-2966 |
|  |  |  | R: ACCATCCTAACACAGACAGTCC |  |
| *Smad2* | NM_001252481.1 | 173 | F: ATGTCGTCCATCTTGCCATTC | 443-615 |
|  |  |  | R: AACCGTCCTGTTTTCTTTAGCTT |  |
| *Smad3* | NM_016769.4 | 636 | F: CCAGCACACAATAACTTGGA | 957-1592 |
|  |  |  | R: AGACACACTGGAACAGCGGA |  |
| *Sox2* | NM_011443.4 | 157 | F: GCGGAGTGGAAACTTTTGTCC | 656-812 |
|  |  |  | R: CGGGAAGCGTGTACTTATCCTT |  |
| *Nanog* | NM_001289828.1 | 364 | F: AGGGTCTGCTACTGAGATGCTCTG | 335-698 |
|  |  |  | R: CAACCACTGGTTTTTCTGCCACCG |  |
| *Oct4* | NM_001252452.1 | 209 | F: TTTCCCTCTGTTCCCGTCAC | 715-923 |
|  |  |  | R: TGATCAACAGCATCACTGAGC |  |
| *Lefty1* | NM_010094 | 109 | F: CAGCTCGATCAACCGCCAGT | 213-321 |
|  |  |  | R: GGCTGGCATGGCTGTGTT |  |
| *Smurf2* | NM_001362894.1 | 196 | F: ATGAAGTCATTCCCCAGCAC | 2431-2626 |
|  |  |  | R: AACCGTGCTCGTCTCTCTTC |  |
| *Thbs1* | NM_001313914.1 | 182 | F: GCAAAGACGTCGATGAGTGC | 2004-2185 |
|  |  |  | R: CGGTTTGCACACCTGTTTGT |  |
| *Acvr2a* | NM_007396.4 | 120 | F: TGAAGCATGAGAACATACTAC | 970-1089 |
|  |  |  | R: GCCTAAGAAAGTCTGACAG |  |
| *Gapdh* | NM_001289726.1 | 275 | F: CCCACTAACATCAAATGGGG | 330-604 |
|  |  |  | R: CCTTCCACAATGCCAAAGTT |  |
